# Integrative morphological, phytochemical, and molecular identification of three invasive and medicinal *Reynoutria* species

**DOI:** 10.1038/s41598-025-90494-2

**Published:** 2025-02-18

**Authors:** Marta Stafiniak, Monika Bielecka, Krzysztof Kujawa, Anna Jezierska-Domaradzka, Bartosz Pencakowski, Aleksander Basiak, Adam Matkowski, Izabela Nawrot-Hadzik

**Affiliations:** 1 Department of Pharmaceutical Biology and Biotechnology, Wroclaw Medical University, Wroclaw, Poland; 2 Statistical Analysis Centre, Wroclaw Medical University, Wroclaw, Poland; 3 Department of Pediatrics, Endocrinology, Diabetology and Metabolic Diseases, Wroclaw Medical University, Wroclaw, Poland

**Keywords:** Natural variation in plants, Plant genetics, Secondary metabolism, PCR-based techniques

## Abstract

The three highly invasive Reynoutria species – *R. japonica* Houtt., *Reynoutria sachalinensis* (F.Schmidt) Nakai, and a hybrid of them – *R.* x *bohemica* J. Chrtek & A. Chrtková are rich sources of biologically active compounds. However, only *R. japonica* is accepted as an herbal drug source in the pharmacopeias as *Polygoni cuspidati rhizoma et radix*, while *R. sachalinensis* is used in folk phytotherapy. The hybrid species can be misidentified as *R. japonica* due to its morphological resemblance. This study intended to explore genetic identity, diversity, and population structure using sequence-related amplified polymorphisms (SRAP) and start codon targeted (SCoT) markers correlated with morphological characteristics and metabolic profiles in fifteen accessions of Reynoutria species from invasive populations in Central Europe (Wroclaw, south-west Poland). The results suggest that morphological identification based on leaf morphology is clear for *R. sachalinensis* but fails to distinguish between *R. japonica* and *R*. × *bohemica* unambiguously. The qualitative HPLC/DAD/ESI-HR-QTOF-MS analysis determined the chemical composition of collected samples, revealing 117 compounds belonging to carbohydrates, stilbenes, flavan-3-ols, procyanidins, anthraquinones, organic acids, and naphthalenes. Out of these, the six compounds were quantitated. Based on LC-MS data for 117 compounds, the cluster analysis categorized the fifteen accessions into two distinctive clusters. All SCoT and SRAP markers showed medium (0.1 to 0.25) to high (0.30 to 0.40) levels of PIC, high levels of polymorphic bands (85 and 89%, respectively) and relatively high mean values for Rp (6.42 and 7.0) and were considered informative and differentiating. Partitioning the genetic diversity with AMOVA showed that variation within the populations was higher than among them, as seen in the combined data from SCoT and SRAP (53.04% vs. 46.96%, respectively) and the analyses of individual markers. Genetic diversity indices revealed that chosen genetic markers efficiently assess the variability within the genus *Reynoutria*. Na varied from 1.57 in *R. sachalinensis* to 3.34 in *R.* x *bohemica*, and Ne was low and almost identical for all species (~ 1.6). Nei’s diversity (H) indicated low genetic diversity (< 0.2) in all species, with the highest value for hybrid (0.184). Despite low H values, Shannon’s index (I) remains high and similar (*R. japonica* − 5.21, *R. x bohemica* – 5.28, and *R. sachalinensis* − 5.45). The number (NPL) and percentage of polymorphic loci (PPL) for the hybrid (160 and 52.98%) were almost twice as high as for the parent species. The populations exhibited moderate G_ST_ values (0.159) and a gene flow (Nm = 1.317). Genetic structure analysis with the Evanno test (ΔK = 2) reveals two genetic groups with the highest admixture in the hybrid population. The cluster analysis dendrograms based on LC-MS data, genetic distance, and leaf morphology categorized the fifteen accessions into three clusters, revealing unequivocal separation of the *R. sachalinensis* from both other taxa. According to Baker’s methodology, the highest correlation between dendrograms was observed between genetic and morphological data, with values of 0.85. Lower correlations were noted between molecular markers and phytochemical profile, as well as between morphology and phytochemical profile. The study suggests the effectiveness of combined morphological, phytochemical, and SCoT and SRAP molecular marker systems in assessing the identity of plant material. This approach can also be used in further studies on the population structure, spread, diversity, and evolution of invasive species such as *Reynoutria* spp.

**Supplementary Information:**

The online version contains supplementary material available at 10.1038/s41598-025-90494-2.

## Introduction

The genus *Reynoutria* Houtt. (*Polygonaceae*) is currently represented by six accepted species (https://powo.science.kew.org/): *Reynoutria ciliinervis* (Nakai) Moldenke, *Reynoutria compacta* (Hook.f.) Nakai, *Reynoutria forbesii* (Hance) T.Yamaz., *Reynoutria japonica* Houtt., *Reynoutria multiflora* (Thunb.) Moldenke, *Reynoutria sachalinensis* (F.Schmidt) Nakai, with two hybrids: *Reynoutria* × *bohemica* Chrtek & Chrtková and *Reynoutria* × *moravica* (Hodálová & Mereďa) Olshanskyi & Antonenko. However, three species are considered noxious invasive weeds in many areas of the world, and these are - *R. japonica* Houtt., *R. sachalinensis* (F.Schmidt) Nakai, and *R. x bohemica* J. Chrtek & A. Chrtková, the latter being a hybrid between two former species. All three have the status of highly invasive alien species in Europe, North America, and New Zealand^1–3^.

*R. japonica* (synonyms: *Polygonum cuspidatum* Sieb. et Zucc.; *Fallopia japonica* (Houtt.) Ronse Decraene *var. japonica;* Japanese knotweed) grows naturally in Sakhalin and Kuril Islands, Japan, Korea, south-west China, Taiwan and Vietnam. It is most often found on sunny slopes of hills and mountains (up to 3800 m above sea level), in river valleys, near roads, and on the edges of forests and it is the main pioneering species inhabiting the areas covered with volcanic lava^1^. The natural range of *R. sachalinensis* (Synonyms: *Fallopia sachalinensis* (F. Schmidt ex Maxim.) Ronse Decraene; *Polygonum sachalinense* F. Schmidt; Giant knotweed) includes Sakhalin, the southern part of the Kuril Islands, Hokkaido and Honshu in Japan, and the island of Ullyng between Japan and Korea. It occurs in similar habitats as the Japanese knotweed but in lower mountain locations – up to 1050 m above sea level^4^. Both species were brought to Europe from Asia as ornamental plants – *R. japonica* in the 1820s and *R. sachalinensis* in the 1860s. They began to appear outside of cultivation just after a few years. Currently, they have taken over Europe, North America, Australia, and New Zealand, and they also occur in North Africa and Chile^5,6^.

*R.* x *bohemica* (synonym: *Fallopia x bohemica* (Chrtek & A. Chrtková) J.P. Bailey; Bohemian knotweed) was first described in Czechia in 1983^7^ as a hybrid formed by pollination of male sterile flowers of *R. japonica* with the pollen of *R. sachalinensis*. It is believed that this hybrid could have appeared shortly after the *R. sachalinensis* was brought to Europe, in the second half of the 19th century^8^. In Asia, where *R. japonica* and *R. sachalinensis* are native species, the hybrid species was only discovered at the end of the 20th century and named *R. x mizushimae* Yokouchi ex T. Shimizu^9^. The late isolation of *R x bohemica* as a separate species was probably due to its misidentification as *R. japonica*. The reason was that the morphological features of both species were similar, within the variability range of *R. japonica*. Caryological studies confirmed the distinctiveness of these species^2,10^.

Knotweeds are enormous rhizomatous perennials that form clonal colonies. The leaves are simple, petiolate, and arranged in two rows on the stems. Size, shape, and leaf hair can serve as diagnostic features to aid in distinguishing the knotweed species^2,11^. Common morphological characteristics are summarized in Table 1. However, the invasive *Reynoutria* species display complex morphological and genetic variations, complicating their accurate identification^2,12^.

**Table 1:** Morphological characteristics of knotweed species based on literature review^3,11–13^.

Feature	*R*. japonica Houtt. (Japanese Knotweed)	*R*. x bohemica Chrtek & Chrtková (Bohemian Knotweed)	*R*. sachalinensis (F. Schmidt) Nakai (Giant Knotweed)
Leaf shape and size	Broadly ovate-triangular, 5–18 cm long, 4–13 cm wide; typically up to 15 cm long and 10 cm wide	Intermediate between *R. japonica* and *R. sachalinensis*; typically broadly ovate, 10–27 cm long, 9–22 cm wide; typically up to 23 cm long and 20 cm wide	Broadly oblong-ovate, heart-shaped or elongated, 15–43 cm long and 10-27 cm wide; typically up to 40 cm long and 25 cm wide length: width ratio c. 1.5
Leaf base	Acuminate/ ovate/ nearly truncate/ truncate/ bluntly wedge-shaped narrowed	Sagittate or moderately cordate; typically base bluntly wedge-shaped (upper leaves) or slightly cordate (lower leaves)	Deeply cordate
Leaf apex/tip	Pointed, cuspidate or acuminate, narrowed into a long tip, curved intermediate	Intermediate, variable pointed, slightly acuminate	Acute, tapering to obtuse/ rounded/ bluntly pointed
Leaf trichomes	Sparse (few to none), short, on leaf underside along veins, reduced to short, single-celled papillae with a strongly swollen base mostly glabrous or scabrous to tuburculate (type A)	Sparse, intermediate trichome shape, density, and length; short, 1-4-celled, with a strongly thickened base (type B)	Dense, longer hairs on leaf underside, 1–5 cells, and 8 or greater (type C)
Stem features	Hollow, green with reddish/purplish/brown speckles and reddish nodes, bamboo-like, up to 2–3 m tall; grow in groups of several to a dozen from a rhizome node (shoot clump)	Variable, often faintly speckled, intermediate height, typically up to 3,5 m; grow in groups of several to a dozen from a rhizome node (shoot clump)	Hollow, robust, usually without speckles, up to 4–4,5 m tall; grow singly or in groups from a rhizome node (shoot clump)
Growth habit	Dense, bamboo-like thickets, upright growth	Combines the dense formation of *R. japonica* with the spreading nature of *R. sachalinensis*	Less dense thickets, spreading growth habit

Rhizomes, less often herb of *R. japonica*, have been used in traditional Chinese and Japanese medicine as an analgesic, antipyretic, expectorant, and diuretic in the treatment of many diseases, including inflammations, infections, asthma, atherosclerosis, hypertension, and heart diseases^14^. The rhizome of *R. japonica* was included in the European Pharmacopoeia as “*Polygoni cuspidati rhizoma et radix*” (Ph. Eur.9)^15^. *R. sachalinensis* and *R. x bohemica* are not considered equivalent medicinal plants. The rhizome of *R. sachalinensis* was traditionally used to some extent as a herbal medicine in East Asia for the treatment of joint pain, jaundice, amenorrhea, cough, burns, scalds, injuries, boils, and wounds^16^, but it was not recognized as a pharmacopoeial raw material. So far, there is no data on the medical use of *R.* x *bohemica*, which has been mainly studied for its biomass production and potential as a biofuel source^17^. According to our previous studies, the rhizomes of these three species differ in their phytochemical profile, which may have an impact on their biological activity^18^. In our previous work, over 130 compounds were tentatively identified from rhizomes of these species belonging to stilbenes, carbohydrates, procyanidins, flavan-3-ols, anthraquinones, phenylpropanoid disaccharide esters, hydroxycinnamic acids, naphthalenes and lignins^19^.

According to the European Pharmacopoeia, the definition of *Polygonum cuspidatum* rhizome and root comprises: “Dried, fragmented rhizome and root, with rootlets removed, of *Reynoutria japonica* Houtt. (syn. *Polygonum cuspidatum* Sieb. et Zucc.) collected in spring or autumn. Content: – emodin (C_15_H_10_O_5_; M_r_ 270.2): minimum 1.0% (dried drug), – polydatin (C_20_H_22_O_8_; M_r_ 390.4): minimum 1.5% (dried drug)”. The determination of emodin and polydatin content according to the European Pharmacopoeia requires the use of two different HPLC methods, which is time-consuming and laborious and may be insufficient in case of misidentification of the source species, especially the hybrid (*R.* x *bohemica*). In previous studies, our research group developed and validated the HPLC-DAD-MS method, which allows the simultaneous determination of six compounds from stilbenes: polydatin and resveratrol; anthraquinones: emodin and physcion as well as sucrose hydroxycinnamic esters: vanicoside A and vanicoside B^18,20^. This analytical method showed significant differences in the content of these compounds between the rhizomes of three *Reynoutria* species (*R. japonica*,* R.* x *bohemica*,* and R. sachalinensis*) which may help distinguish the raw materials. Species distinction is crucial because only *R. japonica* is considered a pharmacopoeial raw material and is often collected from the wild. It is supposed that identification can be difficult with backcrosses of *Reynoutria* species, which may be morphologically similar to *R. japonica*. There is very little data on crossbreeding within the *Reynoutria* spp. and the phytochemical profile of hybrids. However, the probability of their occurrence in the environment is high due to the biology of knotweeds. In Europe, *R. japonica* is present mainly as a single, male-sterile, octoploid genotype, while *R. sachalinensis* and *R. × bohemica* exhibit higher genetic diversity and can reproduce sexually within and between taxa^21^. Both female specimens of *R. japonica* and *R. sachalinensis* can be fertilized with hybrid pollen (*R.* x *bohemica*) yielding seeds from backcrosses.

The development of molecular marker techniques has revolutionized plant genetic studies over the past few decades. Various markers, including RFLPs, RAPDs, AFLPs, ISSRs, SSRs, and SNPs, have gained widespread acceptance due to their stability, cost-effectiveness, and ease of use^22,23^. DNA-based markers have provided higher resolution in detecting genetic variations compared to traditional methods, enabling more precise estimates of genetic differences among individuals and populations^24,25^. The advent of next-generation sequencing has further enhanced marker discovery and application^26^. These molecular tools have proven valuable in various fields, including weed ecology, vector competence studies, and plant breeding^25,27^. Due to the tremendous growth in the availability of public biological databases, the development of functional markers that are located within or near the candidate genes has become considerably easy^28^. This has initiated a shift from random DNA markers to gene-targeted markers, resulting in the creation of novel marker systems such as Start Codon Targeted (SCoT) Polymorphism^29^ and the Sequence-Related Amplified Polymorphism (SRAP) technique^30^.

SCoT markers are designed based on the short, conserved region flanking the ATG start codon in plant genes, while SRAP markers focus on open reading frames (ORFs). Both SCoT and SRAP markers are classified as dominant markers. This means they cannot differentiate between homozygous dominant and heterozygous individuals, only indicating the presence or absence of a particular allele^29,31,32^. Nevertheless, similar to other dominant markers such as random amplified polymorphic DNA (RAPD) and inter simple sequence repeats (ISSR), SCoT and SRAP markers can be employed in genetic analysis, quantitative trait loci (QTL) mapping, and bulk segregation analysis^29^. Recent studies have demonstrated the effectiveness of SCoT and SRAP markers in assessing genetic diversity across various plant species. These markers have shown high polymorphism rates and superior performance compared to other molecular markers like RAPD and ISSR^33–35^. SCoT and SRAP markers have been successfully applied to analyze genetic diversity in safflower^36^, coffee^37^, *Peganum harmala* L.^38^, and *Paris polyphylla* Sm^39^. These markers provide valuable information for germplasm conservation, population genetics, and breeding programs.

SCoT markers were proved to be largely reproducible. The factors influencing SCoT marker reproducibility include primer GC content and 3’-end stability, rather than primer length or annealing temperature^40,41^. Additionally, SCoT markers have demonstrated higher resolution and variability in detecting genomic information than RAPD markers^33,34^.

Numerous studies have demonstrated that SRAP is highly effective for genetic diversity analysis, cultivar identification, and phylogenetic studies^42–44^ but like other dominant markers, they do not distinguish between different zygosity states^45,46^. Ferriol et al.^42^ found that the information provided by SRAP markers aligned more closely with morphological variability and the evolutionary history of the morphotypes than that provided by AFLP markers. Additionally, SRAP markers have shown higher effectiveness in several studies, as evidenced by their higher polymorphism information content in safflower^36^. Overall, SCoT and SRAP markers offer robust tools for genetic diversity analysis across various plant species.

The objectives of this study were to (1) analyze the morphological characteristics of these populations, (2) assess genetic variation and population structure across fifteen *Reynoutria* populations, (3) evaluate the chemical profiles of all individuals, and (4) determine the relationships between leaf morphology, genetic polymorphism, and chemical diversity within and among populations.

## Results

### Morphological analysis of *Reynoutria* specimens

**Fig. 1:**
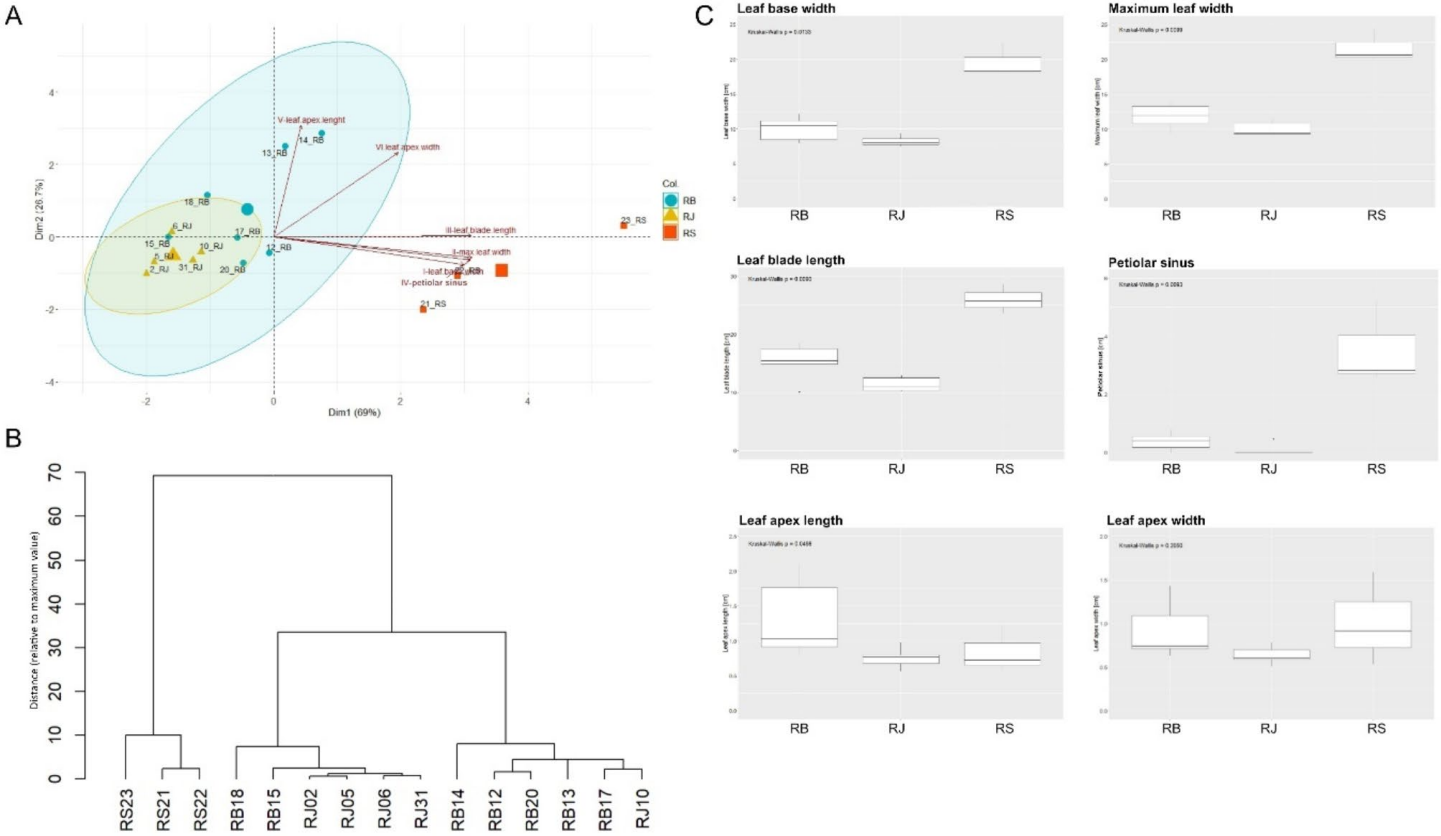
Morphological analysis of 15 *Reynoutria* accessions using six numerical leaf characters. Principal component analysis (PCA) with loadings (**A**); Dendrogram (**B**); Boxplots of the variability of morphological characters between the three taxa. The boxes represent medians, Q1, and Q3, and whiskers show data ranges excluding outlying data (**C**).

The morphological variation of 15 knotweed accessions was assessed in 150 middle-stem leaves (Supplementary Table 1) using a morphometric analysis, which has been considered the most reliable and widely used for knotweed identification^13,47,48^. Seven leaf characters were recorded for each specimen, out of which six were included in principal component analyses (PCA) and dendrogram construction (Fig. 1). The PCA was performed on datasets comprising morphological leaf characters related exclusively to the shape and size of the leaf lamina, excluding indumentum traits. Based on these six numerical characters, the analysis aimed to determine whether leaf shape and size alone are sufficient for identifying knotweed taxa, as trichome morphology is not readily observable in the field without a sufficiently magnifying glass.

Both PCA and dendrogram showed similar results (Fig. 1A and B). *R. sachalinensis* formed a distinct cluster, although data were too scarce to draw the ellipse. The hybrid *R. × bohemica* exhibited the greatest range of variation and was closely aligned with its parent taxon, *R. japonica.* The two latter taxa were not distinctly separated, resulting in overlapping clusters (Fig. 1A).

Hierarchical cluster analysis with the Euclidean distance measure and Ward’s clustering method constructed from morphological data grouped the fifteen *Reynoutria* individuals into three clusters (Fig. 1B). Notably, only the Giant Knotweed (represented by RS21, RS22, and RS23) formed a distinct branch. Despite a clear tridirectional structuring of the three taxa, noticeable overlaps were also present on the dendrograms. Morphologically similar *R. japonica* and *R. × bohemica* were not reliably separated. While they formed two clusters, individuals within them did not consistently correspond to a single species.

Loading plots and boxplots presenting six selected characters employed for the morphological analysis show that the dimensions vary between species (Fig. 1A and C). The parameters most effectively distinguishing *R. sachalinensis* from other species are leaf blade length and width. In contrast, the differentiation of *R. japonica* from *R. × bohemica* requires additional morphological parameters, as these species exhibit significant similarity in leaf characteristics. Accurate distinction between these species is achievable only when trichome morphology is also employed for analysis (Supplementary Table 1). Sample 15, although a hybrid, notably displays typical *R*. *japonica* morphology. Thus the most appropriate characters for separating knotweeds are leaf base width, leaf lamina width, leaf length, and petiolar sinus. According to Kruskal-Wallis analysis, only the apex width and length do not show statistically significant variation (Fig. 1C). Among the three species, *R*. *japonica* consistently has the smallest leaf dimensions. It is evident, that also the majority of leaves growing in the middle part of the stem exhibit considerable morphological variation. This is particularly visible in *R. × bohemica*, where leaves from the same stem can differ to such an extent that one may resemble the typical morphology of RJ, while another displays characteristics typical of RB.

Presented result suggests that morphological analysis alone is insufficient for the clear differentiation of *R. japonica* and *R. × bohemica*.

### Genetic analysis of *Reynoutria* specimens

A total of thirty-six SCoT primers and sixty-six SRAP primer combinations were screened, out of which eighteen primers (Table 8) were able to amplify reproducible bands. These primers were employed to analyze genetic diversity and population structure.

To analyze the variability of *Reynoutria* spp. specimens, 18 sets of primers were used to study the SCoT and SRAP banding patterns across the populations. Nine selected SCoT primers produced 145 bands (16.11 bands per primer), of which 125 were polymorphic. Similarly, nine SRAP primer combinations produced 152 bands, with 135 being polymorphic (16.89 bands per primer). The percentage of polymorphic bands ranged from 70 to 91% for SCoT primers, with an average of 85%, and from 81 to 95% for SRAP primers, with an average of 89%. Among the SCoT primers, SCoT1 exhibited very high polymorphism and the highest resolving power (12.5). For the SRAP primers, the SRAP43 pair showed the highest polymorphism (95%), while SRAP32 had the highest resolving power (Rp) at 9.88, which strongly correlates with the ability to distinguish between genotypes. All of the SCoT and SRAP primers scored medium (0.1 to 0.25) to high (0.30 to 0.40) levels of polymorphism information content (PIC) (Table 2), which is considered differentiating for dominant markers.

**Table 2:** Informativeness parameters of SCoT and SRAP primers for fifteen analyzed *Reynoutria* accessions.

Marker	Total number of bands	Number of polymorphic bands	% Poly-morphism	Resolving power (Rp)	Mean PIC value	Marker	Total number of bands	Number of polymorphic bands	% Poly-morphism	Resolving power (Rp)	Mean PIC value
SCoT1	22	20	0.91	12.5	0.352	SRAP11	15	14	0.93	8.25	0.349
SCoT2	19	17	0.89	7.38	0.262	SRAP15	19	17	0.89	7.13	0.271
SCoT7	16	12	0.75	3.88	0.177	SRAP22	16	13	0.81	6.25	0.267
SCoT13	18	16	0.89	7.38	0.290	SRAP23	15	13	0.87	5.13	0.241
SCoT15	10	7	0.7	2.88	0.202	SRAP25	17	15	0.88	7.75	0.306
SCoT16	17	15	0.88	7.63	0.306	SRAP32	19	17	0.89	9.88	0.340
SCoT24	13	11	0.85	5.75	0.300	SRAP35	16	15	0.94	5.5	0.235
SCoT26	16	13	0.81	4.88	0.229	SRAP43	20	19	0.95	8.63	0.302
SCoT32	14	14	1	5.5	0.277	SRAP45	15	12	0.8	4.5	0.226
Total	145	125	-	57.75	-	Total	152	135	-	63	-
Mean	16.11	13.89	0.85	6.42	0.27	Mean	16.89	15	0.89	7.00	0.28

Genetic diversity indices (Table 3) revealed that chosen genetic markers efficiently assess genetic variability within the genus *Reynoutria*. The average observed number of alleles per locus (Na) varied from 1.57 in Sakhalin knotweed to a relatively high 3.34 in bohemian knotweed, and an effective number of alleles (Ne) was low and almost identical for all species (~ 1.6). Nei’s gene diversity (H) indicated low genetic diversity (< 0.2) in all species with the highest value for hybrid (0.184). H values were similar for *R. japonica* and *R. sachalinensis* (0.101 and 0.106 respectively). Despite low H values, Shannon’s Information index (I) remains similar, with high values for *R. japonica* (5.21), *R. x bohemica* (5.28), and *R. sachalinensis* (5.45). The number of polymorphic loci (NPL) and percentage of polymorphic loci (PPL) for the hybrid (160 and 52.98%) were almost twice as high as for the parent species. The populations exhibited moderate Nei’s coefficient of gene differentiation (G_ST_ = 0.159) and a gene flow (Nm = 1.317). The AMOVA test showed that genetic variation observed within the populations was statistically significantly (*p* < 0.001) higher than variance among populations, as seen in both the combined data from SCoT and SRAP (53.04% vs. 46.96%, respectively) and in the analyses of individual markers (Table 4).

**Table 3:** Summary of genetic variation statistics in fifteen accessions of three *Reynoutria* species with combined two different molecular markers (SRAP and SCoT).

Population	Observed number of alleles (Na)	Effective number of alleles (Ne)	Gene diversity/ expected heterozygosity (H)	Shannon’s information index (I)	Number of polymorphic (NPL)	% of polymorphic loci (PPL)	Nei’s coefficient of gene differentiation (G_ST_)	Gene flow (Nm)
*R. japonica*	2.60	1.63	0.101	5.21	84	27.81		
*R.* x *bohemica*	3.34	1.59	0.184	5.28	160	52.98		
*R. sachalinensis*	1.57	1.64	0.106	5.45	85	28.15		
Total							0.159	1.317

**Table 4:** Analysis of molecular variance (AMOVA) using SRAP and SCoT molecular markers in *Reynoutria* Spp.

Source of variation	df	SCoT + SRAP			SCoT			SRAP		
		MS	Est. var	Var	MS	Est. var	Var	MS	Est. var	Var
Among population	2	151.31399	24.43719	46.96035	72.51190	11.73301	47.22186	78.80208	12.70418	46.72138
Within population	13	27.60073	27.60073	53.03965	13.11355	13.11355	52.77814	14.48718	14.48718	53.27862
Total	15	44.09583	52.03792		21.03333	24.84656		23.06250	27.19136	
		Phi = 0.4696035, *p* < 0.001			Phi = 0.4722186, *p* < 0.001			Phi = 0.4672138, *p* < 0.001		

**Fig. 2:**
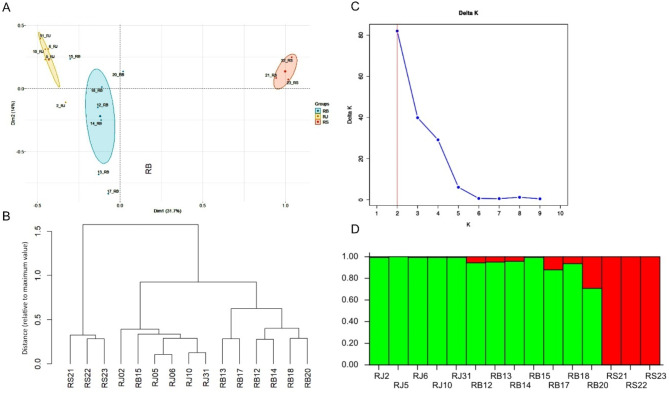
Genetic relationships and genetic structure of *Reynoutria* accesions based on combined SRAP and SCoT markers. Multiple correspondence analysis (MCA) (**A**); dendrogram for binary data (**B**); estimation of the best number of ΔK (**C**); population structure for K = 2 (**D**).

To better represent the relationship among the tested *Reynoutria* specimens, multiple correspondence analysis (MCA) and cluster analysis was performed based on combined SCoT and SRAP data (Fig. 2). The MCA (Fig. 2A) and dendrograms (Fig. 2B) revealed species-specific clustering of different knotweed genotypes. The cluster analysis dendrogram (Fig. 2B) categorized the samples into three clusters based on all polymorphic SRAP and SCoT fragments. While the dendrogram distinctly separates *R. sachalinensis* from the other species, *R. × bohemica* and *R. japonica* individuals exhibit considerable similarity. Although they form separate clusters, their close genetic relationship is evident, as seen in sample RB15, which is positioned within the *R. japonica* cluster. The UPGMA results were relative to the centers of similarity. The MCA analysis (Fig. 2A) revealed that the sample RJ2 was the only individual that diverged from the genetically cohesive *R. japonica* cluster, showing a shift towards *R*. × *bohemica*. *R. sachalinensis* formed a distinct and well-defined cluster, clearly separated from the other individuals. The hybrid group exhibited the greatest genetic diversity. Samples RB12, RB14, and RB18 were positioned closest to the center of similarity, while RB15 demonstrated a strong genetic affinity with *R. japonica*. In contrast, RB20 displayed a slight tendency towards *R. sachalinensis*, and RB13 and RB17 markedly differed from the other hybrid samples.

Following Structure Selector analysis, the maximum ΔK for the SCoT and SRAP marker data were observed at K = 2, and the 15 accessions, predefined into three populations, were grouped into two genetic groups (K = 2) (Fig. 2C). The Bayesian bar plot analysis revealed a clear genetic distinction for *R. sachalinensis* (red bars), while *R. japonica* formed a separate and well-defined genetic group (green bars) (Fig. 2D). Although a third distinct cluster was not identified, *R. × bohemica* accessions, centrally positioned in the bar plot, exhibit genetic admixture from both parental species, with a predominance of *R. japonica* (indicated by a more significant proportion of green color in the bars). These findings align with the cluster analysis dendrogram and MCA analysis, further confirming the distinct genetic structure of *R. sachalinensis* and the high genetic similarity between *R. japonica* and *R. × bohemica*, with the hybrid being the most genetically diverse.

### HPLC/DAD/ESI-HR-TOF-MS analysis

To determine the composition of the collected *Reynoutria* samples, qualitative HPLC/DAD/ESI-HR-QTOF-MS analyses were performed. The UHPLC-QTOF-MS analysis revealed a total of 117 detected compounds (Table 5) that belong to carbohydrates, stilbenes, flavan-3-ols, procyanidins, anthraquinones, organic acids, and naphthalenes. Among the 117 detected compounds, 26 remained unassigned and without a clear indication of their phytochemical nature and two were tentatively defined as carbohydrates.

**Table 5:** UHPLC-QTOF-MS analysis revealed a total of 117 different compounds.

No.	Compound	Rt. [min]	UV max [nm]	m/z [M-H]-	Error (ppm)	Ion formula	MS^2^ Main-Ion (relative intensity %)	MS^2^ fragments (relative intensity %)	References
1	Unknown carbohydrate	1.05	ND	341.1090	−0.1	C12H21O11	113.0246(100)	101(58), 119(42), 179(21)	HMDB0000258
2	Unknown carbohydrate	1.07	ND	683.2247	0.4	C24H43O22	341.0401(100)		-
3	Hexoside derivative of caffeic acid	1.08	220, 228, 280	377.0857	5,6	C18H17O9	341,1096(100)	179(70), 119(43), 161(23), 101(15), 149(12), 215(7), 377(5)	^49^
4	Unknown	1.09	ND	439.0815	4.5	C26H15O7	96.9632(100)	241(2)	-
5	Unknown	1.1	ND	195.0508	2.0	C6H11O7	-	-	-
6	Organic acid e.g., citric acid	1.13	ND	191.0197	0	C6H7O7	111.0086(100)	112(6)	HMDB0000094
7	Organic acid e.g. malic acid	1.15	ND	133.0143	−0.3	C4H5O5	115.0026(100)		HMDB0000156
8	Procyanidin dimer, Type B	1.6–1.8	225, 280	577.1349	0.5	C30H25O12	289.0723(100)	407(58), 125(34), 425(16), 451(10), 161(10), 245(8)	^50^
9	Vanillic acid 4-beta-D-glucoside	1.81	225, 280	329.0872	1.7	C14H17O9	209.0451(100)	167.0350(56), 239.0560(20), 125.0243(15), 179.0348(12)	HMDB0302469
10	Procyanidin trimer, Type B	1.8-2.0	225, 280	865.198	1.8	C45H37O18	575.1100(100)	287(92), 575(90), 865(75), 425(58), 289(45), 695(44), 713(42)	^50^
11	Catechin	2.18	225, 280	289.0717	0.2	C15H13O6	123.0451(100)	109(95), 221(71), 203(64), 151(57), 245(32)	^50^
12	Procyanidin dimer, Type B	2.2–2.4	225, 280	577.1349	0.4	C30H25O12	289.0721(100)	407(75), 125(42), 425(21), 161(14)	^50^
13	Epicatechin	2.8–2.9	225, 280	289.0717	0.2	C15H13O6	123,0451(100)	109(91), 203(69), 221(63), 151(61), 137(40), 245(32)	^50^
14	Unknown	3.25	280	499.1293	2.3	C18H27O16	499.1298(100)	97(10), 111(6)	-
15	Procyanidin trimer, Type B	3.36	225, 280	865.1977	0.8	C45H37O18	577.1341(100)	287(82), 575(75), 865(66), 425(54), 713(44), 413(42), 695(40)	^51^
16	Unknown	3.42	220, 284	383.0066	−5.6	C18H7O10	285.0407(100)	125.0241(62), 303.0508(30), 275.0562(23), 177.0193(21)	
17	Piceatannol glucoside	3.5	220, 290, 318	405.1187	0.9	C20H21O9	243.0671(100)	244(15), 293(4), 275(2)	^51^
18	Peperomic ester	3.6	220, 282, 325	395.0985	−0.3	C18H19O10	215.0352(100)	189.0558(12), 259.9113(3)	^52^
19	Unknown	3.6	282, 325	439.1085	1.8	C16H23O14	439.1089(100)	97(21), 424(2)	-
20	Resveratrolside	3.9	220, 304, 315	389.123, 435.1291[M + COO]-	1.2	C20H21O8	227.0713(100)	228(16), 225(9), 185(2)	^51^
21	Procyanidin dimer monogallate	4.1	225, 280	729.1449	1.6	C37H29O16	407,0776 (100)	289(62), 577(45), 441(29), 451(27)	^51^
22	Unknown	5.39	220, 275	269.0124	4.9	C7H9O11	189.0556 (100)		-
23	Piceid	6.1	220, 304, 315	389.1245	−0.8	C20H21O8	227.0716(100)	228(13), 185(2)	^51^
24	Epicatechin-3-O-gallate	6.3	220, 279	441.0825	0.6	C22H17O10	169.0145(100)	289(51), 125(22), 245(14)	^51^
25	Procyanidin dimer, Type B	6.4	225, 280	577.1345	1.2	C30H25O12	289.0717(100)	407(59), 125(38), 425(18), 161(14), 451(9), 245(8)	^19^
26	Procyanidin trimer monogallate	6.7–6.8	225, 280	1017.2127	−3.1	C52H41O22	729.1431(100)	577(30), 865(29), 287(27), 441(19)	^51^
27	Piceatannol glucoside isomer	7.3	220, 290, 318	405,1187	0.9	C20H21O9	-	-	-
28	Resveratrol hexoside	7.8	220, 304, 315	389.1238	1.1	C20H21O8	227.0713(100)	228(15), 185(1), 143(0.5)	^51^
29	Resveratrol derivative	8.4	220, 282, 325	431.1356	−2.0	C22H23O9	227.0722(100)	316(3), 185(2)	^51^
30	Resveratrol hexoside	8.7	220, 304, 315	389.1249	−1.9	C20H21O8	227.0718(100)	228(15), 185(2), 143(0.5)	^51^
31	Unknown	8.9	220, 280	505.1125	3.0	C27H21O10	289.0716(100)	189.0559(26), 215.0348(23), 125.0247(11)	
32	Procyanidin dimer monogallate e.g.Procyanidin dimer b2 3’-*O*-gallate	9.0	225, 280	729.1533	−2.3	C37H29O16	-	-	HMDB0037966, ^19^
33	Aloesone hexoside	9.03	220, 280, 420	393.1188	0.8	C19H21O9	231.0663(100)	232(13), 257(2), 187(0,5)	^51^
34	Emodin-glucoside	9.8	221, 269, 281, 423	431.0979	1.1	C21H19O10	431,0977(100)	269(87), 240 (9)	^51^
35	Lapathoside D	10	220, 290, 315	633.1821	0.7	C30H33O15	145,0295(100)	487(43), 633(30)	^51^
36	Unknown	10.57	220, 280	311.0224	−8.7	C16H7O7	231.0664(100)	174.9560(8), 274.8814 (6)	
37	Resveratrol	10.7	220, 306, 319	227.0714	−0.5	C14H11O3	143.0501(100)	185(71), 227(35), 183(17), 159(12), 117(6)	^51^
38	N-trans-feruloyltyramine	11.3	220, 281, 323	312.1242	−0.5	C18H18NO4	148.0529(100)	178.0515(51), 190.0514(25), 174.9566(23), 274.9909(20)	HMDB0029365, ^19^
39	Trihydroxy-methoxy-flavone	11.7	220, 282	299.0557	1.4	C16H11O6	256.0378(100)	299(46), 284(3)	^53^
40	Torachrysone- hexoside	12.3	226, 266, 325	407.1359	−1.1	C20H23O9	245.0845(100)	246(14), 230(11)	^51^
41	Resveratrol-isomer	12.80	220, 306, 319	227.0714	−0.5	C14H11O3	-	-	-
42	Emodin-glucoside	12.87	221, 269, 281, 423	431.099	−1.5	C21H19O10	269.0465(100)	431(50), 311(5)	^51^
43	Phenylpropanoid-derived disaccharide ester, e.g. Helonioside B	12.96	220, 280	735.2141	0.2	C34H39O18	-	-	^19^, PubChem CID: 21,636,195
44	Emodin bianthrone-hexose-(malonic acid)-hexose	13.0	220, 280, 325	919.2318	−1.7	C45H43O21	458.1215(100)	416(49), 671(18), 713(14), 875(13)	^19^
45	Unknown	13.3	220, 280	345.1554	3.5	C32H50O16	-	-	
46	Emodin-8-O-(6’-O-malonyl)-glucoside	14.2	220, 281, 423	517.0991	−0.7	C24H21O13	473.1098(100)	269(68), 311(3)	^51^
47	Unknown Torachrysone derivative	14.4	220, 280, 320	449,1457	−0.9	C22H25O10	245.0818(100)	246(14)	^51^
48	Sulfonyl torachrysone	14.6	220, 312	325.0395	−2.3	C14H13O7S	245.0829(100)	230 (34)	^54^
49	Lapathoside C	14.7	220, 283, 315	809.2291	0.9	C40H41O18	-	-	^19^, PubChem CID: 11,061,786
50	Tatariside E	14.9	220, 283, 315	717.2032	0.6	C34H37O17	-	-	^19^, PubChem SID: 275,763,001
51	Physcionin/Rheochrysin	15.0	221, 272, 423	445.1136	0,9	C22H21O10	283.0610(100)	284(17), 307(5)	HMDB0040511/ HMDB35931
52	MS^2^ Fragment of Physcionin/Rheochrysin, e.g. physcione	15.1	221, 272, 423	283.0614	−0.7	C16H11O5	-	-	PubChem CID: 10,639
53	Emodin bianthrone-hexose-(malonic acid)-hexose	15.5	220, 281, 325	919.2318	−1.7	C45H43O21	458.1215(100)	416(49), 671(18), 713(14), 875(13)	^51^
54	Tatariside A	15.6	220, 285, 315	759.2128	1.8	C36H39O18	-	-	^19^, PubChem SID: 275,762,997
55	Hydropiperoside	15.7	222, 298, 313	779.2188	0.7	C39H39O17	779.2176(100)	145(80), 633(61), 453(6), 615(5)	^51^
56	Unknown	15.8	220, 280, 315	285.0402	0.9	C15H9O6	285.0406(100)	241.0509(11), 257.0456(7)	
57	Unknown	16.10	220, 282, 318	485.2004	2.7	C23H33O11	-	-	
58	Emodin bianthrone-hexose-(malonic acid)-hexose	16.12	220, 280, 320	919.2318	−1.7	C45H43O21	458.1215(100)	416(49), 671(18), 713(14), 875(13)	^51^
59	(3,6-O-di-*p*-coumaroyl)-*β*- fructofuranosyl-(2→1)-(2’-*O*-acetyl-6’-*O*-feruloyl)-*β*- glucopyranoside*	16.13	220, 298, 315	851.2420	−1.9	C42H43O19	851.2423(100)	145.0290(25), 705.2010(13), 809.2309(6), 675.2019(3)	^18,19^
60	Hydropiperoside	16.14	222, 298, 313	779.2193	−1.4	C39H39O17	779.2176(100)	145(80), 633(61), 453(6), 615(5)	^19^
61	Phenylpropanoid-derived disaccharide ester, e.g. Smiglaside C	16.2	220, 280, 317	819.2349	0.5	C38H43O20	-	-	^19^, PubChem CID: 139,292,105
62	Hydropiperoside B	16.3	220, 280, 330	1027.2156	−2.0	C50H43O24	-	-	^19^
63	Derivative of Emodin bianthrone-hexose- malonic acid	16.4	220, 280, 320	1005.2322	−1.6	C48H45O24	-	-	^19,51^
64	Unknown	16.5	220, 280, 320	329.2322	3.5	C18H33O5	-	-	
65	Tatariside A isomer	16.7	220, 285, 315	759.2159	−2.2	C36H39O18	-	-	^19^
66	Unknown physcion derivative	17.0	220, 275, 423	1063.2325	3.4	C50H47O26	283.0607(100)	325(13), 487(7)	^51^
67	MS^2^ Fragment of Unknown physcion derivative	17.0	221, 272, 423	283.0614	−0.7	C16H11O5	-	-	
68	Emodin bianthrone-hexose-(malonic acid)-hexose	17.1	220, 280, 325	919.2309	−0,6	C45H43O21	458.1211 (100)	416(50), 502(23), 713(18), 671(18), 875(10)	^19,51^
69	Unknown	17.3	220, 286	299.0189	2,9	C15H7O7	299.0188(100)	255(31), 211(19), 227(12)	
70	Vanicoside C*	17.5	222, 298, 315	821.2315	−2	C41H41O18	821.2309(100)	145(34), 675(15), 453(3)	^18,19^
71	Derivative of Emodin bianthrone-di-hexose	17.6	220, 285, 325	1019.2444	1.8	C49H47O24	458.1211(100)	502(23), 931(9), 460(4), 416(2)	^51^
72	Hydropiperoside B	17.7	220, 280, 330	1027.2156	−2.0	C50H43O24			^19^
73	Phenylpropanoid- derived disaccharide esters	18.0	220, 298, 315	1151.3422	−1.8	C59H59O24	1151.3358(100)	955(20), 145(5), 1133(2), 1103(2), 1009(2), 809(2)	^51^
74	Emodin isomer	18.1	221, 288, 315, 430	269.0453	2.5	C15H9O5	269.0467(100)	225.0551(30), 241.0501(18)	
75	Emodin derivative	18.2	220, 286, 316	349.0026	6.5	C11H9O13	269.0460(100)		
76	Vanicoside B isomer	18.9	222, 298, 315	955.2668	−0.2	C49H47O20	955.2651(100)	956(37), 145(22), 809(18), 957(17), 453(1)	^51^
77	Hydropiperoside isomer	19.0	222, 298, 313	779.2188	0.7	C39H39O17	779.2176(100)	145(80), 633(61), 453(6), 615(5)	^19^
78	Unknown	19.2	220, 280, 330	801.2236	1.5	C38H41O19	-	-	^19^
79	Tatariside B*	19.25	220, 298, 315	893.2515	−0.6	C44H45O20	-	-	^18,19^
80	Vanicoside B*	19.4	222, 298, 315	955.2662	0.4	C49H47O20	955.2696(100)	957(17), 809(15), 145(14), 453(1)	^18,19^
81	Lapathoside A	19.5	220, 290, 315	985.2768	0.4	C50H49O21	-	-	^19^, PubChem CID: 10,011,201
82	Emodin bianthrone-hexose, e.g. Ramosin	19.72	220, 283, 325	671.1769	0.2	C36H31O13	-	-	^19^, PubChem CID: 363,268
83	Diacetyl derivative of (diacetoxy-methoxyphenyl) acroyl-*O*-*p*-coumaroyl-*O*- caffeoylquinic acid	19.76	220, 288, 325	861.2493	−3.9	C40H45O21	-	-	^19^
84	Emodin bianthrone- hexose-malonic acid	19.78	220, 280, 325	757.1766	1.1	C39H33O16	458.1220(100)	502(8), 713(5), 254(4), 416(2)	^19^
85	Derivative of Emodin bianthrone-di-hexose	19.8	220, 285, 325	1019.2477	−1.4	C49H47O24	-	-	^19^
86	Vanicoside B isomer	20.0	222, 298, 315	955.2662	0.4	C49H47O20	-	-	
87	Questin	20.27	222, 286, 313, 430	283.0604	2.8	C16H11O5	240.0425(100)	268(2)	^51^
88	Dihydroferuloyl vanicoside B	20.31	220, 290, 315	1133.3331	−2.6	C59H57O23	-	-	^19^
89	Emodin bianthrone- hexose-malonic acid	20.5	220, 280, 325	757.1766	1.1	C39H33O16	458.1220(100)	502(8), 713(5), 254(4), 416(2)	^19^
90	Vanicoside A*	21.2	222, 298, 315	997.2741	1.8	C51H49O21	997.2776(100)	145(8), 851(5), 821(1)	^18,19^
91	Methyl derivative of Emodin bianthrone-hexose	21.3	220, 283, 325	685.1900	3.9	C37H33O13	-	-	^19^
92	Emodin bianthrone-hexose e.g. Ramosin isomer	21.42	220, 283, 325	671.1769	0.2	C36H31O13	-	-	^19^
93	Tatariside C	21.43	220, 290, 315	935.2638	−2.4	C46H47O21	-	-	^19^, PubChem SID: 275,762,999
94	Emodin bianthrone- hexose-malonic acid	21.8	220, 283, 325	757.1766	1.1	C39H33O16	458.1220(100)	502(8), 713(5), 254(4), 416(2)	^51^
95	Emodin bianthrone-hexose e.g. Ramosin isomer	21.9	220, 283, 325	671.1769	0.2	C36H31O13	-	-	^19^
96	Methyl derivative of Emodin bianthrone-hexose- malonic acid	22.0	220, 283, 325	771.1936	−0.8	C40H35O16	-	-	^19^
97	Emodin bianthrone- hexose-malonic acid	22.5	220, 283, 325	757.1766	1.1	C39H33O16	458.1220(100)	502(8), 713(5), 254(4), 416(2)	^51^
98	Methyl derivative of Emodin bianthrone-hexose	22.8	220, 283, 325	685.1900	3.9	C37H33O13	-	-	^19^
99	Unknown	23.9	220, 280, 325	311.0552	3.0	C17H11O6	-	-	
100	Methyl derivative of Emodin bianthrone-hexose- malonic acid	24.0	220, 283, 325	771.1936	−0.8	C40H35O16	-	-	^19^
101	Methyl derivative of Emodin bianthrone-hexose- malonic acid	24.6	220, 283, 325	771.1936	−0.8	C40H35O16	-	-	^19^
102	Unknown	25.0	220, 283, 325	194.0823	−0.9	C10H12NO3	-	-	
103	Methyl derivative of Emodin bianthrone-hexose- malonic acid	25.1	220, 283, 325	771.1936	−0.8	C40H35O16	-	-	^19^
104	Emodin*	25.4	221, 248, 267, 288, 430	269.0464	−3.1	C15H9O5	269.0464(100)	225(29), 241(10), 197(2), 181(1)	^18,19,51^
105	Unknown	27.1	-	311.1688	5.9	C13H27O8	-	-	
106	Unknown	27.4	220, 280, 325	285.0402	1.0	C15H9O6	285.0404(100)	257.0455(22), 241.0515(9)	
107	Emodin bianthrone/Reidin B	28.3	221, 280, 360	509.1239	0.6	C30H21O8	254.0578(100)	-	HMDB0038509
108	Unknown	28.6	-	325.1854	4.3	C14H29O8	-	-	
109	Unknown	28.7	-	295.2271	2.5	C18H31O3	-	-	
110	Unknown	28.8	220, 280, 360	625.3443	5.3	C47H45O	-	-	
111	Emodin bianthrone/Reidin B isomer	28.9	221, 280, 360	509.1239	0.6	C30H21O8	254.0578(100)	-	HMDB0038509
112	Unknown	29.0	-	291.0657	1.8	C18H11O4			
113	Unknown	30.1	225, 280, 325	297.1864	−1.4	C20H25O2	297.1864(100)	281.1540(7), 232.9242(5)	
114	Unknown	30.2	220, 360	311.2022	−1.7	C21H27O2	149.0976(100)	150.1006(11), 174.9556(7)	
115	Unknown	30.3	227, 280, 363	523.1405	−1.3	C31H23O8	254.0593(100)		
116	Unknown	30.6	-	325.2159	4.3	C22H29O2	-	-	
117	Unknown	30.7	220, 375	387.1560	10.8	C25H23O4	387.1564(100)	-	

*New compound reported for the first time in *Reynoutria* rhizomes.

**Fig. 3:**
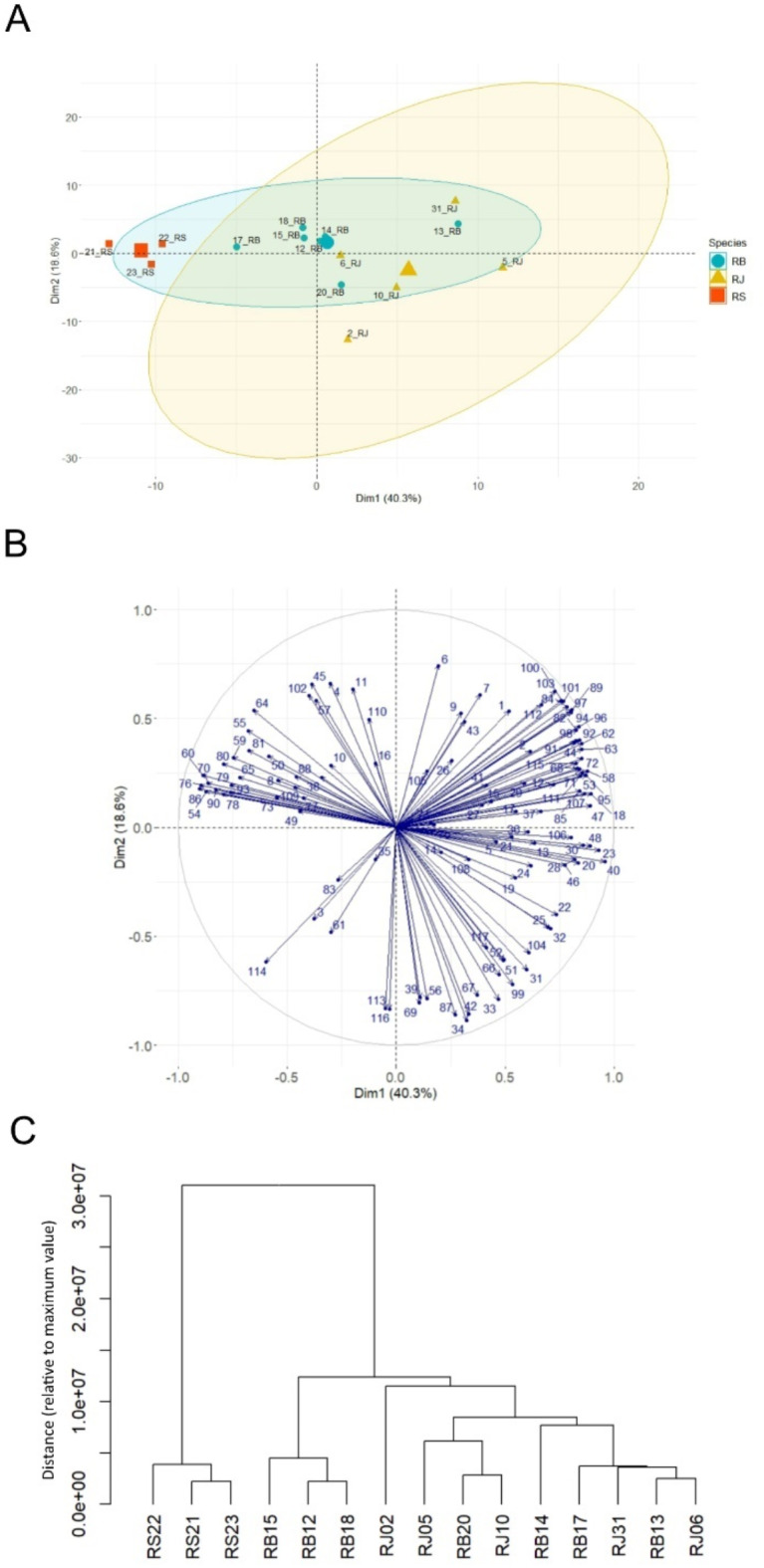
Metabolic relationships among 15 *Reynoutria* genotypes based on LC-MS data for 117 compounds. Principal components analysis (PCA) plots indicating the general grouping of the variables in the data sets of extracts. The scores plot was computed using the first two principal components (PC1–40.3% vs. PC2-18.6%). Cumulative variance = 91.65988% for 8 components. (**A**); Loading plot of PCA results obtained from LC-MS data. Numbers represent the compounds listed in Table 5 (**B**); Dendrogram constructed based on LC-MS data for 117 compounds (**C**).

Principal component analysis (PCA) was carried out using LC-MS data (peak intensities) acquired from 15 tested specimens. The visualization of the PCA scores plot shows similarities/dissimilarities between (PC1) and within (PC2) sample clusters. On the PCA scores plot (Fig. 3A) all samples identified as *R. sachalinensis* (RS21, RS22, RS23) were located on the left side of the plot, while almost all samples identified as *R. japonica* were located on the right side of the plot. According to the loading plot (Fig. 3B) compounds furthest to the left and right of the area are most responsible for this differentiation. The biggest impact on the created *R. sachalinensis* cluster was demostrated for phenylpropanoid derived disaccharide esters such as compund 60 (Hydropiperoside), 70 (Vanicoside C), 76 (Vanicoside B isomer), 86 (a Vanicoside B isomer), 54 (Tatariside A), 90 (Vanicoside A), 80 (Vanicoside B), 79 (Tatariside B), 65 (a Tatariside A isomer), 59 ((3,6-O-di-*p*-coumaroyl)-*β*- fructofuranosyl-(2→1)-(2’-*O*-acetyl-6’-*O*-feruloyl)-*β*- glucopyranoside), 93 (Tatariside C) present in these samples in the highest quantity. On the other hand, *R. japonica* has been influenced by many other compounds (located most to the right in the loading plot), out of which the most important are naphthalene derivatives, anthraquinones and stilbenes: 40 (a Torachrysone- hexoside), 48 (Sulfonyl torachrysone), 23 (Piceid), 47 (an unknown torachrysone derivative), 18 (a Peperomic ester), 95 (Emodin bianthrone-hexose), 53 (Emodin bianthrone-hexose-(malonic acid)-hexose), 58 (Emodin bianthrone-hexose-(malonic acid)-hexose), 85 (a Derivative of Emodinbianthrone-di-hexose), 107 (Emodin bianthrone), 30 (a Resveratrol hexoside), 20 (Resveratroloside), 28 (a Resveratrol hexoside).

The samples from *R.* x *bohemica* are closer to the center of the PCA score plot, indicating that phenylpropanoid derivatives as well as naphthalenes, anthraquinones and stilbenes had comparably significant effect on their distribution between the left and right sides of the plot (Fig. 3A). However, it can be noted that two samples are located much further from the center. Sample RB17 is located closer to *R. sachalinensis*, which clearly differs in terms of chemical composition from sample RB13, located far from the center on the right side of the area. Dissimilarities between the samples distributed on the left or right side of the PCA scores plot are explained by PC2. Most of the *R. japonica* samples are located in the fourth quadrant, and their position was influenced by such compounds as 34 (Emodin-glucoside), 42 (Emodin-glucoside), 87 (Questin), 33 (Aloesone hexoside). Sample RJ2 is located at the lowest in the fourth quadrant, indicating the significant content of these compounds, which differentiates it from the other *R. japonica* samples. Sample RJ31 is clearly on the opposite side from RJ2, indicating significant differences between them. The position of sample RJ31 was most influenced by compounds located in the third quadrant. Most of the *R.* x *bohemica* samples are in the second and third quadrants of the PCA chart, with the exception of RB20. A position near the center of the plot indicates a similar influence of compounds or a slight influence of compounds distributed on the edges of the loading plot.

PCA analysis showed that only samples from *R. sachalinensis* formed a separate, distinct cluster. Among *R.* x *bohemica* and *R. japonica* specimens, samples stood out from the rest, making it impossible for them to form separate clusters.

Similarly, the cluster analysis dendrogram (Fig. 3C) categorized the samples into two distinctive clusters based on LC-MS data for 117 compounds. As in the PCA analysis, only samples from *R. sachalinensis* formed a separate cluster, with other samples being clustered together and categorized into subclusters formed by a mix of RB and RJ samples.

### HPLC/DAD/ESI-HR-TOF-MS quantitative analysis

Quantitative HPLC/DAD/ESI-HR-TOF-MS analysis was conducted for 15 *Reynoutria* specimens. The abundance of six compounds such as: piceid, resveratrol, vanicoside A, vanicoside B, emodin and physcion was determined in the rhizomes of analyzed *Reynoutria* samples (Table 6).

**Table 6:** Mean (of six replicates) substance content in mg per 1 g of dry extract of rhizomes.

Compound/sample	RJ2	RJ5	RJ6	RJ10	RJ31	RB12	RB13	RB14	RB15	RB17	RB18	RB20	RS21	RS22	RS23
Piceid	36.98	72.87	26.40	36.18	33.55	19.69	26.19	25.53	8.76	10.84	22.70	12.16	0.00	0.00	0.00
SD for piceid	2.34	1.76	1.89	1.97	1.14	1.36	0.52	1.21	1.18	0.95	2.48	0.99	0.00	0.00	0.00
Resveratrol	1.66	1.35	0.40	0.76	1.65	0.98	0.59	1.13	0.18	0.29	1.49	0.50	0.00	0.00	0.00
SD for resveratrol	0.10	0.05	0.04	0.04	0.07	0.05	0.02	0.08	0.03	0.02	0.35	0.07	0.00	0.00	0.00
Vanicoside A	0.44	0.30	0.80	0.21	0.33	0.76	0.17	0.81	0.37	1.16	1.01	0.67	2.31	0.71	1.16
SD for vanicoside A	0.05	0.05	0.06	0.03	0.04	0.09	0.02	0.15	0.04	0.12	0.15	0.13	0.32	0.12	0.12
Vanicoside B	4.70	4.37	7.12	2.53	5.39	9.12	1.91	8.40	4.97	11.07	9.83	5.57	14.73	6.70	10.01
SD for vanicoside B	0.26	1.04	0.55	0.33	0.55	1.02	0.18	1.47	0.39	0.61	1.52	0.99	1.80	0.96	0.51
Emodin	13.30	5.97	2.07	4.24	2.07	1.92	2.06	2.71	0.77	2.77	1.70	4.64	0.55	0.34	0.71
SD for emodin	0.70	0.20	0.08	0.32	0.07	0.14	0.06	0.22	0.10	0.14	0.49	0.47	0.01	0.02	0.06
Physcion	8.03	6.93	2.16	4.02	2.09	2.12	3.46	3.63	1.03	2.90	1.90	5.67	0.62	0.53	0.52
SD for physcion	0.45	0.60	0.07	0.88	0.14	0.15	0.22	0.27	0.14	0.25	0.65	1.05	0.05	0.03	0.03

Calibration curves as well as area and other calculations are presented in the supplement. SD, standard deviation.

**Fig. 4:**
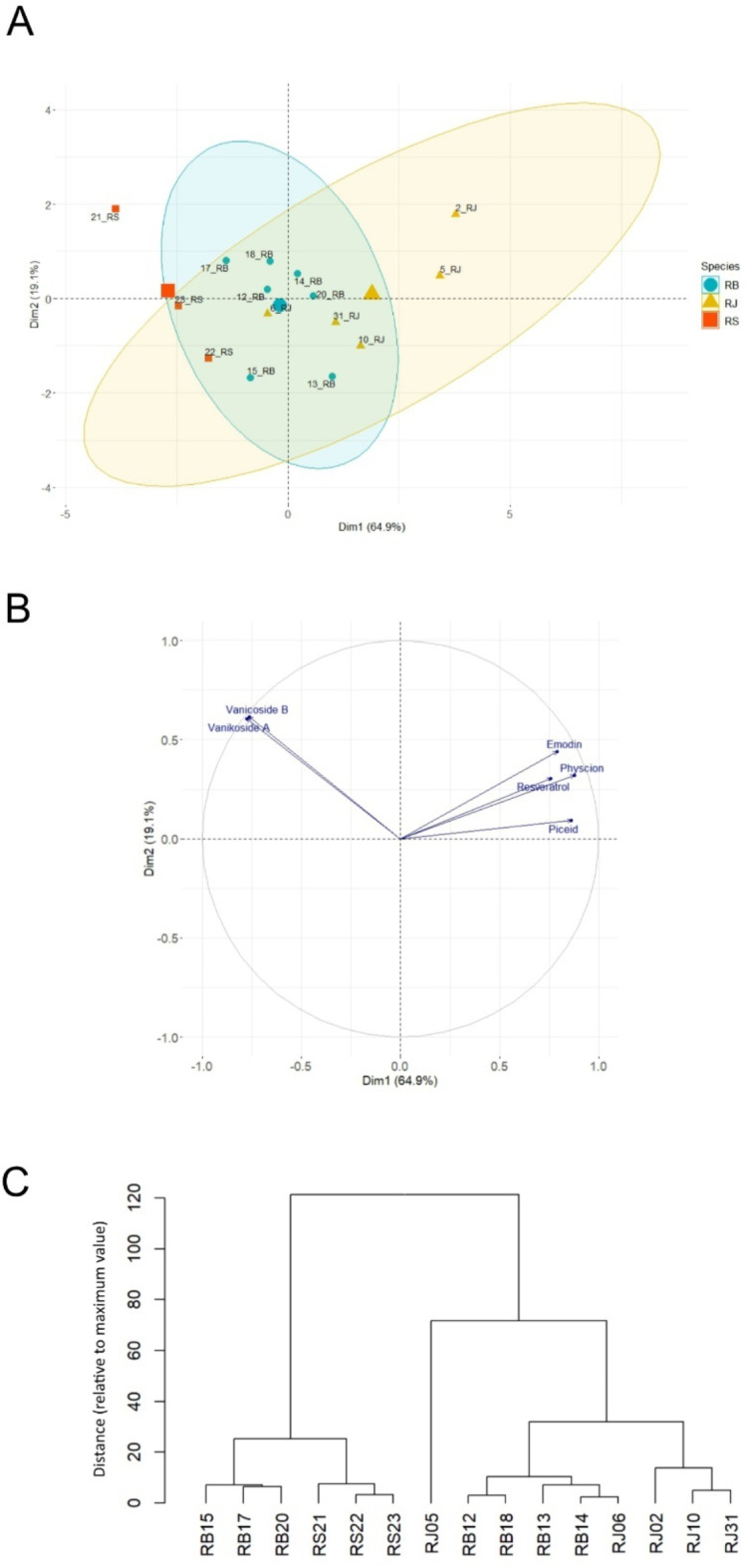
Metabolic relationships among 15 *Reynoutria* genotypes based on quantitative analysis of six compounds. Principal components analysis (PCA) plots indicating the general grouping of six variables in the data sets of extracts. The scores plot was computed using the first two principal components (PC1–64.9% vs. PC2–19.1%) (**A**); Loading plot of PCA results obtained from LC-MS data for six quantified compounds. (**B**); Dendrogram constructed based on LC-MS data for six quantified compounds (**C**).

The content of six quantified compounds clearly differs between samples from *R. sachalinensis* and *R. japonica*. *R.* x *bohemica*, on the other hand, contains their intermediate quantities. The most abundant compound turned out to be piceid, which has been found exclusively in rhizomes of *R. japonica* and *R. bohemica*. In the rhizomes of R. *sachalinensis*, piceid and resveratrol were below the detection level. Despite of that, neither the PCA analysis nor the dendrogram constructed with the six quantified compounds, did not allowed for the unequivocal differentiation between analyzed *Reynoutria* accessions (Fig. 4).

Sample RJ06 is more similar to other *R.* x *bohemica* samples, probably due to its relatively low resveratrol, piceid, emodin and physcion content and relatively high vanicoside A and B content (Table 6). The opposite trend is observed in sample RB13, where very low content of vanicosides was observed, which makes this sample similar to other *R. japonica* samples.

## Discussion

Recent studies have deepened our understanding of the taxonomy and identification of *Reynoutria* species, revealing significant challenges due to their complex morphological, cytological, and genetic diversity. Morphological analysis alone is often insufficient for accurately distinguishing species, primarily due to extensive genetic and phenotypic variability^55^. For instance, *R. × bohemica* exhibits intermediate traits between its parent species but resembles *R. japonica*^10,56^. These variations are critical in understanding invasion dynamics and management strategies^57,58^.

Key morphological features used for differentiation include leaf shape, trichome characteristics, and base/apex morphology. Japanese knotweed has broadly ovate leaves with a straight or slightly rounded base and sharply pointed apex. Giant knotweed features heart-shaped leaves with a deeply heart-shaped base and pointed ends, while Bohemian knotweed shows intermediate traits, often making it difficult to distinguish without detailed examination. Hybrids can be recognized by intermediate leaf and stand size, slightly cordate leaf bases, and variations in trichome morphology^8^. Leaf indumentum has been suggested as the most reliable distinguishing feature^10^. However, there is no single trait that would allow for clear differentiation between taxa^2,10,11^. Furthermore, the utility of morphological traits diminishes in regions with diverse hybrid populations, where morphological characteristics often overlap, complicating identification^59^. The collection site of studied plants (Fig. 6) is one of many regions in Europe, where cross-pollination between different taxa of *Reynoutria* occurs producing varied populations of hybrids.

While anatomical characteristics can help differentiate some species^60^, hybridization and polyploidy further complicate identification^61,62^. Although cytological studies have revealed varying ploidy levels across the genus, there is no consistent correlation between ploidy and morphological traits^11^. *R. japonica* is typically octoploid, *R. sachalinensis* ranges from tetraploid to hexaploid, and *R. × bohemica* is generally hexaploid^2,11^. Hybridization and introgression events further increase genetic complexity^12,59^. Cytogenetic analysis can be challenging due to the small, undifferentiated chromosomes, requiring advanced techniques like fluorescence in situ hybridization (FISH) for accurate analysis^63^.

Genetic diversity studies in *Reynoutria* species reveal intricate patterns. While early research indicated low diversity in invasive *R. japonica* populations in Europe^62,64^, recent genomic analyses suggest considerable genetic variation within this taxon in North America^65^. In contrast, *R. sachalinensis* and the hybrid *R. × bohemica* consistently exhibit higher genetic variability across studies^62,64,65^. This diversity is likely shaped by multiple introductions and hybridization events^65^. Genetic diversity within *Reynoutria* has been further elucidated using markers such as SNP, nuclear genes *LEAFY*, and isozyme analyses, revealing significant variability, especially in hybrids^57,59,66^. These approaches reveal cryptic genetic diversity and complex hybridization dynamics in invasive *Reynoutria* populations, underscoring the need for further research to elucidate the genetic mechanisms underlying their invasive success. This diversity is closely tied to the species’ invasive potential and regeneration abilities^66^. Despite progress in genetic studies, differentiating hybrids with varying ploidy levels remains difficult^67^.

Chemical profiling, particularly using high-performance separation methods like liquid or gas chromatography, has long been a valuable tool in distinguishing of species and infraspecific variants such as chemotypes, chemical races or to detect admixtures and adulterations. Additionally, using LC-MS data processing and analysis platforms, often available online and free of charge, chemical profiling can become fast, reliable and useful for species identification. Moreover, this identification method is advantageous especially for the authentication of pharmacopoeial raw material, such as the rhizomes, which are separated from the aerial parts and often dried and where the information about other morphological features is missing.

In *Reynoutria* species HPLC and LC-MS analytical studies revealed distinct phytochemical compositions^18,19^. In this work, we have demonstrated that *R. sachalinensis* is distinguished by the impact of phenylpropanoid disaccharide esters such as hydropiperoside, vanicosides, tatarisides, and their isomers (Fig. 3B). This observation is consistent with our earlier studies, where a significantly higher content of these compounds was demonstrated in *R. sachalinensis* than in *R. japonica* and *R*. x *bohemica*^18,19^. We have also proved that *R. japonica* separates mainly by a higher content of naphthalene derivatives, anthraquinones and stilbenes (Fig. 3B). Such findings support the role of chemical profiling as an important complementary tool to morphological and genetic analyses. However, some other fingerprinting methods, such as HPTLC did not allow to clearly distinguish these three taxons as in Bensa and co-workers who compared flavan-3-ol profiles using several mobile and stationary phases on HPTLC plates^68^. As it was shown, all three species had similar qualitative profiles and only quantitative differences were observed. Thus, sole phytochemical fingerprinting is insufficient to obtain reliable discrimination between the hybrid and the parent species.

To address the limitations of traditional morphological analysis, combining chemical profiling, cytogenetics, and genetic techniques such as targeted fingerprinting offers a more comprehensive framework for species identification. Polymorphism Information Content (PIC) and resolving power (Rp) are key metrics used to assess the effectiveness of genetic markers. PIC, first introduced by Botstein et al.^69^ for linkage studies, is nowadays widely used in genetic diversity and genome-wide association studies to assess marker usefulness^70^ as it corresponds to the markers’ ability to detect polymorphisms among individuals in a population^71^. Higher PIC values reflect a greater capacity for distinguishing between genotypes. For dominant markers, the maximum PIC value is 0.5^72–74^. PIC analysis has practical applications in various fields, including plant breeding and conservation genetics, as demonstrated in studies on *Citrullus colocynthis* in Iran^75^. Several equations exist for calculating PIC, with variations for dominant and co-dominant markers^71^. Allele frequencies influence PIC values and can vary between breeds within a species^76^. In this study, all of the SCoT and SRAP primers scored medium (0.1 to 0.25) to high (0.30 to 0.40) levels of PIC which is considered differentiating for dominant markers (Table 2) and makes them a suitable tool for studying genetic diversity. Resolving power (Rp) measures a marker system’s discriminatory ability by calculating the number of genotypes that can be separated based on specific markers within a population^77^. High PIC and Rp values reflect greater marker efficiency and informativeness in population genetics. Both, PIC and Rp metrics are thus crucial for assessing the efficiency, effectiveness, and potential of marker systems in population genetics and molecular biology.

The number of bands produced per primer also serves as an indicator of marker informativeness. Higher band production correlates with an increased ability to detect polymorphisms, which is crucial in distinguishing genotypes and cultivars^78^. In our study, SCoT1 exhibited the highest polymorphism and resolving power (12.5). For the SRAP primers, the SRAP43 pair showed the highest polymorphism (95%), while SRAP32 had the highest resolving power (Rp) at 9.88. The percentage of polymorphic bands ranged from 70 to 91% for SCoT primers, with an average of 85%, and from 81 to 95% for SRAP primers, with an average of 89%, which is considered relatively high (Table 2).

Low genetic diversity in all analyzed species (expressed as Nei’s gene diversity index H < 0.2) suggests their limited genetic variation and potential vulnerability to environmental changes. The low H values in invasive species indicate they may have undergone a genetic bottleneck, possibly due to clonal reproduction, limited introductions, or strong selection pressures^79,80^. The hybrid shows higher genetic diversity (H = 0.184) than both parent species (H = 0.101 and 0.106), which is expected due to hybridization. Increased heterozygosity in hybrids can provide greater adaptability and fitness, potentially explaining its invasive success^81,82^. The lower genetic diversity within *R. japonica* and *R. sachalinensis* populations may limit adaptability to changing environments, making them more reliant on phenotypic plasticity rather than genetic variation. Shannon’s Information Index (I) is correlated with Nei’s gene diversity but generally provides a more comprehensive view by incorporating allelic frequency distribution. Shannon’s index values for *R. japonica* (I = 5.21), *R.* × *bohemica* (I = 5.28), and *R. sachalinensis* (I = 5.45) indicate high genetic diversity across all analyzed species. Shannon’s index accounts for both genetic richness and evenness, meaning higher values suggest more significant genetic variation within populations. These results indicate that while Nei’s gene diversity suggests genetic uniformity, Shannon’s index might highlight the presence of rare alleles or higher complexity among studied accessions, particularly, rather than widespread variation. The number of observed alleles (Na) suggests moderate to high diversity in *R.* × *bohemica* (3.34) but lower diversity in *R. japonica* (2.60) and *R. sachalinensis* (1.57). The rather low effective number of alleles (Ne = ~ 1.6) reveals constrained genetic diversity across all species, indicating that allelic richness exists, but allele frequencies are not evenly distributed. NPL and PPL values confirm that *R.* × *bohemica* exhibits significantly higher genetic diversity than its parent species, likely due to hybridization. In contrast, the relatively low genetic diversity in *R. japonica* and *R. sachalinensis* suggests clonal reproduction and possible genetic bottlenecks, which may limit their evolutionary potential^79^. Since Nei’s coefficient of gene differentiation (G_ST_ = 0.159) is on the boundary between moderate and high^83^, populations exhibit some genetic distinctiveness while still maintaining shared genetic variation. Gene flow (Nm) can decrease local adaptation by homogenizing populations that grow in different habitats^84^. For studied *Reynoutria* populations, Nm = 1.317 means the gene flow is still occurring but not at a high enough rate to homogenize populations completely. The moderate to high genetic differentiation and gene flow observed in *Reynoutria* populations align with the species’ primary mode of vegetative reproduction. Vegetative reproduction, such as clonal growth through rhizomes, typically limits genetic variation within individual clones but can allow for some genetic exchange between geographically separated individuals or populations, primarily through mechanisms like pollen transfer or seed dispersal^85,86^. The observed moderate differentiation suggests that although populations are primarily clonal, genetic diversity is maintained, likely due to occasional sexual reproduction and limited gene flow. This gene flow rate prevents the complete isolation of species through genetic connectivity and allows for their local adaptation and differentiation.

**Fig. 5:**
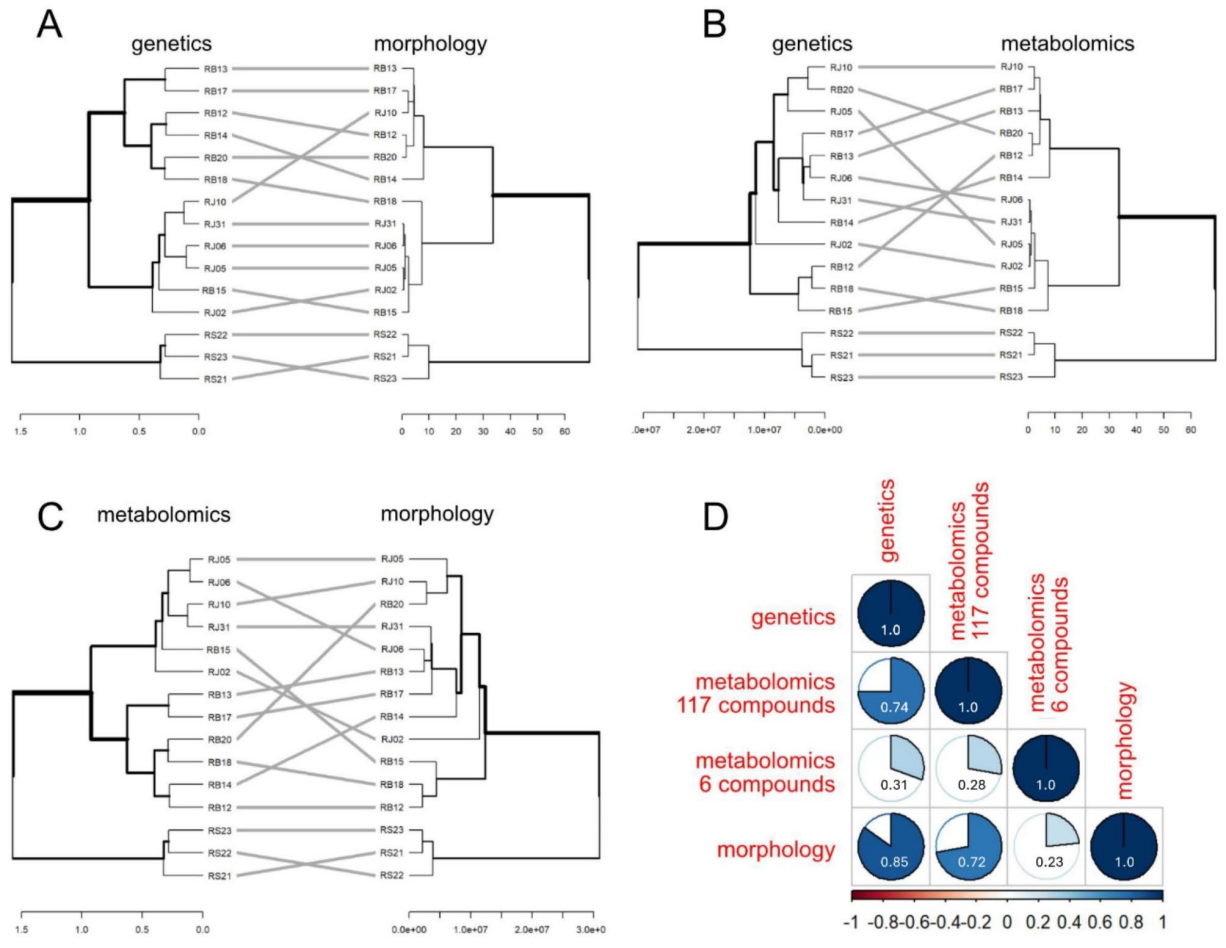
Correlation analysis of dendrograms in *Reynoutria* generated using genetic, metabolomics (117 compounds), and morphological data sets. Comparison of genetic and morphological dendrograms (**A**), genetic and metabolomic dendrograms (**B**), metabolomic and morphological dendrograms (**C**), and graphical representation of Baker’s correlation coefficient for genetic, morphological, and metabolomic (117 and 6 compounds) dendrograms (**D**).

In this work, cluster analysis dendrograms were created to categorize the *Reynoutria* samples based on their morphological traits (Fig. 1B), chemical composition (Figs. 3C and 4C), and genetic diversity (Fig. 2B). Correlations between all dendrograms are presented in Fig. 5.

The highest correlation between dendrograms was observed between genetic and morphological data, with values of 0.85 (according to Baker’s methodology) (Fig. 5D). The genetic dendrogram corresponded with the morphological dendrogram analysis but confirmed the problematic affiliation of several specimens (Fig. 5A). For example, sample RB15 aligns closely with *R. japonica* in both genetic and morphological analyses. However, sample RJ10, despite being morphologically ambiguous and clustering with *R. × bohemica*, is genetically a typical *R. japonica*. This does not prove a genetic method’s inability but implicates the possibility of cross-breeding between hybrids and one of the parental organisms, though there is no doubt that Sakhalin knotweed is a distinct clade, morphologically chemically and genetically.

Lower correlations were noted between genetics and metabolomics, as well as between morphology and metabolomics. The genetic and metabolomic dendrogram (Fig. 5B) comparison supports the distinct clustering of *R. sachalinensis*. Chemically, RB15 shows more similarities to *R. japonica*, and RJ10 reaffirms its genetic link to *R. japonica*, despite its morphological discrepancies.

The greatest inconsistencies among the comparative analyses arose from the metabolomic and morphological comparisons (Fig. 5C). While *R. sachalinensis* was distinctly differentiated, attempts to correlate the remaining individuals with their respective species were confusing. Moreover, the analysis based on only six key chemical compounds proved insufficient, making it impossible to establish a meaningful dendrogram correlation.

Morphological markers are visual indicators that were often reported insufficient in raw material identification. Chemical profiling has long been a valuable tool in distinguishing of species, however a sole phytochemical fingerprinting has been proven unsatisfactory to obtain reliable discrimination between the hybrid and parent species. Unlike plant genetic features, both, plant morphology and plant metabolome are phenotypic characteristics resulting from the activity of particular genes and influenced throughout the plant life by various environmental factors.

## Conclusions

This study explored genetic identity, diversity, and population structure using SRAP and SCoT markers correlated with morphological characteristics and LC-MS-based phytochemical profiles in three invasive *Reynoutria* species. The comprehensive investigation supported the combined approach’s effectiveness in assessing the origin of plant material, especially the identity of the pharmacopeial herbal drug *Polygoni cuspidate rhizome et radix* (*R. japonica* rhizomes).

It has also been shown that phytochemical differences between *R.* × *bohemica* and *R. japonica* are less significant, in contrast to *R. sachalinensis.* Therefore, the rhizome raw material from *R. × bohemica* may be suitable for medicinal use, as they share comparable chemical properties with *R. japonica.* This supports the potential application of *R. × bohemica* as an abundant source of bioactive constituents or as a whole herbal drug with limitations regarding specific pharmacopoeial guidelines.

Morphologically similar plants may not be genetically related and the profile of bioactive phytochemicals may be entirely dissimilar. Therefore, a characterization of molecular features should be included in recommended identification procedures to minimize the risk of mistakes or unexpected pharmacological properties. Moreover, the complex identification and discrimination methods can aid in field studies and assessing spread, diversity and evolution of the invasive species, which is a prerequisite for sustainable environmental management.

## Methods

### Plant material

**Fig. 6:**
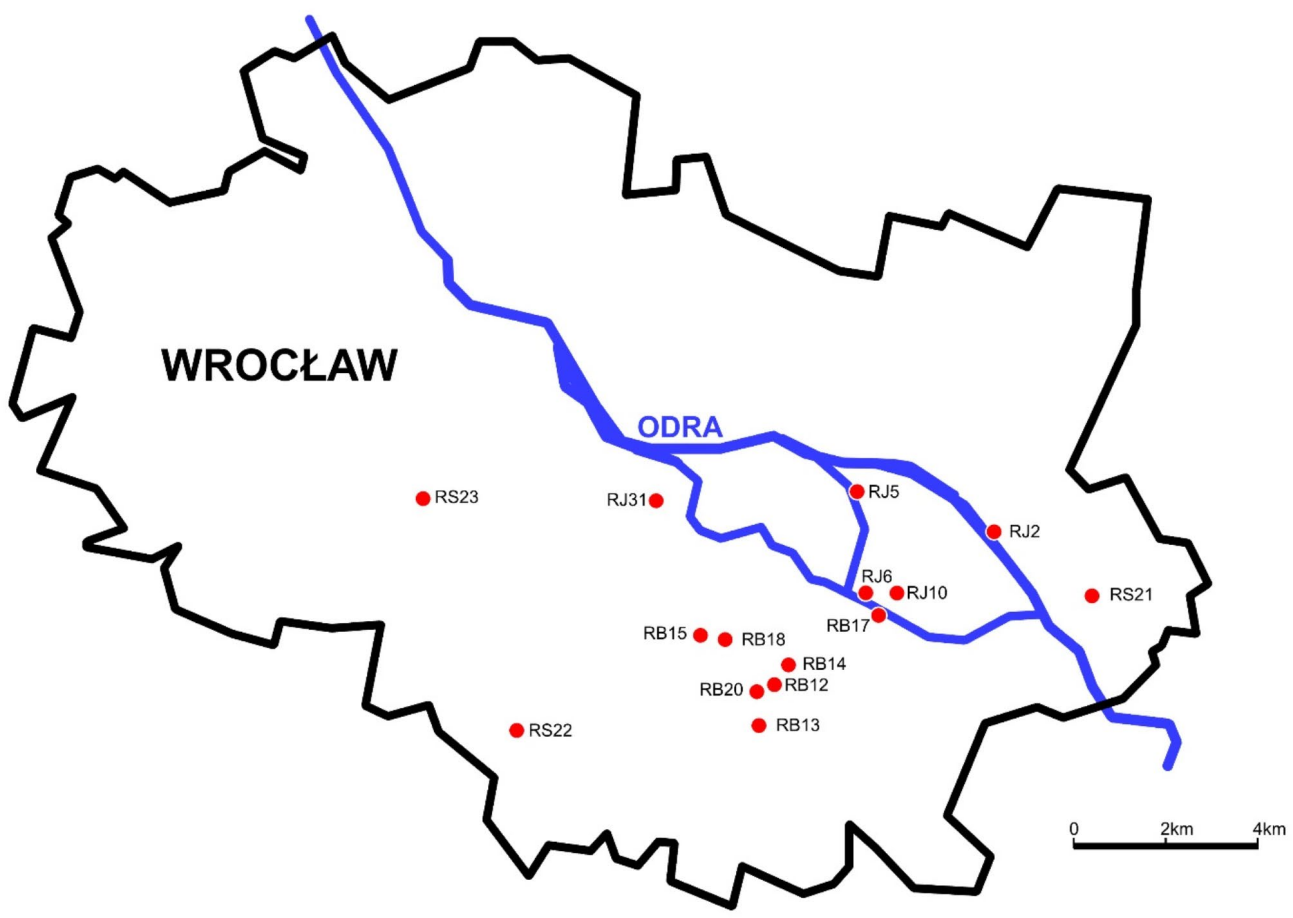
Map showing the geographic location of fifteen sampling locations. The map has been created using Affinity Photo (1.10.6).

Our study investigated three invasive knotweed taxa collected in the urban environment of Wroclaw, Poland. The samples were collected from fifteen distinct populations across various geographical locations, and they were classified based on their leaf shape and size, trichome type and morphology^12^. To evaluate the patterns of genetic variation in the *Reynoutria* genus, leaf material was obtained from a total of 15 plant individuals representing: five *R. japonica*, seven *R. × bohemica*, and three *R. sachalinensis* populations. Rhizomes from the same specimens were collected for phytochemical analysis. Voucher specimens of all taxa have been deposited at the Herbarium of the Botanical Garden of Medicinal Plants, Wroclaw Medical University (Poland). Figure 6; Table 7 detail the collected information for examined samples.

**Table 7:** Geographical characteristics of *Reynoutria* spp. accessions used in this study.

No.	Sample name	Coordinates	Species	Voucher specimen	Collection date
1	2RJ	51°06.945’ N 17°06.787’ E	*R. japonica*	P-129	10/2021
2	5RJ	51°07.404’ N 17°04.146’ E	*R. japonica*	P-130	10/2021
3	6RJ	51°06.168’ N 17°04.311’ E	*R. japonica*	P-131	10/2021
4	10RJ	51°06.233’ N 17°04.957’ E	*R. japonica*	P-133	10/2021
5	31RJ	51°07.304’ N 17°00.455’ E	*R. japonica*	P-134	10/2021
6	12RB	51°05.153’ N 17°02.681’ E	*R*. x *bohemica*	P-135	10/2021
7	13RB	51°04.671’ N 17°02.390’ E	*R*. x *bohemica*	P-136	10/2021
8	14RB	51°05.383’ N 17°02.932’ E	*R*. x *bohemica*	P-137	10/2021
9	15RB	51°05.739’ N 17°01.295’ E	*R*. x *bohemica*	P-138	10/2021
10	17RB	51°06.035’ N 17°04.643’ E	*R*. x *bohemica*	P-139	10/2021
11	18RB	51°05.666’ N 17°01.746’ E	*R*. x *bohemica*	P-140	10/2021
12	20RB	51°05.080’ N 17°02.378’ E	*R*. x *bohemica*	P-141	10/2021
13	21RS	51°06.190’ N 17°08.635’ E	*R*. *sachalinensis*	P-142	10/2021
14	22RS	51°04.617’ N 16°57.792’ E	*R*. *sachalinensis*	P-143	10/2021
15	23RS	51°07.342’ N 16°56.085’ E	*R*. *sachalinensis*	P-144	10/2021

The plants investigated in this study have been collected by the botanic garden staff who act according to the Ministry of Environment, decision number: DOPozgiz-4210-26-6024-05/KL. The plants have been formally identified by the Botanic Garden of Medicinal Plants curator, Klemens Jakubowski MSc.

### Morphological analysis

Only plants exceeding 1.5 m in height were included in the study. Ten fully mature, mid-branch leaves were collected from each plant (in the middle of the stem the shape of the leaves is most characteristically developed). Leaf blade length and width were measured as overall indicators of size. Leaf apex morphology (length and width) and leaf base width and shape (petiolar sinus) were also examined. Trichome morphology was assessed at 100x magnification and categorized into three classes based on trichome height, cell number and basal cell morphology following descriptions by Gammon et al. and Zika et al.^12,47^. Based on these observations, each sample was identified as *R. sachalinensis*, *R.* × *bohemica*, or *R. japonica* according to Table 1 criteria. Individuals not fully matching parental traits were labeled as *R.* × *bohemica*. Hierarchical cluster analysis was conducted on morphological traits using the R packages ‘stats’ r core^87^ and ‘dendextend’^88^, employing the Euclidean distance measure and Ward’s clustering method. The Kruskal-Wallis test was used to evaluate the statistical significance of morphological data.

### Genomic DNA isolation

Young leaves from each of the 15 plant individuals were collected and dried for 3 h at 55 °C. After grinding in liquid nitrogen, total DNA was extracted using the Plant/Fungi DNA Isolation Kit (Norgen Biotek). The DNA concentration was measured with a NanoDrop™ 2000 spectrophotometer (Thermo Scientific™). The quality of DNA was estimated by agarose gel electrophoresis.

### SRAP and SCoT PCR amplification

All PCR reactions were performed within a total volume of 20 µl containing genetically engineered thermophilic Hybrid DNA polymerase (EURx, Poland), approximately 50 ng of genomic DNA, and 0.5 µmol of each primer (Sigma-Aldrich, USA). The primer sequences, parameters, and combinations of markers are illustrated in Table 8. Both, SRAP and SCoT markers were amplified by gradient Bio-Rad T100 Thermal Cycler (Bio-Rad, USA). The amplicons were separated on 1.5% agarose gels using Perfect™ 100 bp DNA Ladder as standard (EURx, Poland) in a Tris Acetate-EDTA (TAE) buffer at 7 V/cm, stained with ethidium bromide and visualized under UV light with G: BOX Chemi XRQ gel doc system (Syngene, UK).

Initially, 66 SRAP primer combinations were used to amplify 15 genomic DNA templates of *Reynoutria* spp. From those, nine primer combinations: me1 + em1 (SRAP11), me1 + em5 (SRAP15), m32 + em2 (SRAP22), me2 + em3 (SRAP23), me2 + em5 (SRAP25), me3 + em2 (SRAP32), me3 + em5 (SRAP35), me4 + em3 (SRAP43), me4 + em5 (SRAP45) were selected as they provided the greatest reproducibility and the highest number of bands per gel (Table 2).

**Table 8:** The list of all primers used in studies.

SRAP primer name	Sequence (5’→3’)	SCoT primer name	Sequence (5’→3’)
Forward		SCoT1	CAACAATGGCTACCACCA
me1	TGAGTCCAAACCGGATA	SCoT2	CAACAATGGCTACCACCC
me2	TGAGTCCAAACCGGAGC	SCoT7	CAACAATGGCTACCACGG
me3	TGAGTCCAAACCGGAAT	SCoT13	ACGACATGGCGACCATCG
me4	TGAGTCCAAACCGGACC	SCoT15	ACGACATGGCGACCGCGA
me5	TGAGTCCAAACCGGAAG	SCoT16	ACCATGGCTACCACCGAC
Reverse		SCoT24	CACCATGGCTACCACCAT
em1	GACTGCGTACGAATTAAT	SCoT26	ACCATGGCTACCACCGTC
em2	GACTGCGTACGAATTTGC	SCoT32	CCATGGCTACCACCGCAC
em3	GACTGCGTACGAATTGAC		
em4	GACTGCGTACGAATTTGA		
em5	GACTGCGTACGAATTAAC		
em6	GACTGCGTACGAATTGCA		

SRAP forward and reverse primers based on Li et al.^30^ and SCoT primers selected from the list of 36 proposed by Collard et al.^29^.

According to the primer sequences designed by Collard and Mackill^29^, all of the 36 SCoT primers were synthesized and tested and nine primers with clearly separated bands, stable amplification, and rich polymorphism were selected for further analysis (Table 2).

Due to the polymerase modification, the optimal reaction conditions differed from standard SCoT and SRAP PCR protocols^29,30^. SRAP amplification thermal cycling conditions were as follows: 30 s of initial denaturing at 98 °C, 35 cycles of 10 s at 98 °C, 30 s at 35 °C for the five initial cycles, and 50 °C for the next 30 cycles, and 2 min at 72 °C; this was followed by a final extension of 7 min at 72 °C. SCoT PCR amplifications (as it is a single primer reaction) were programmed as follows: an initial denaturation step at 98 °C for 30 s followed by 35 cycles of 98 °C for 10 s, 50 °C for 30 s, and 72 °C for 2 min; the final extension was at 72 °C for 7 min.

### Molecular data analysis

The molecular analysis of SRAP markers was done according to the procedure described by Li and Quiros^30^ with some modifications. All SRAP primer combinations were initially screened using a group of all 15 samples^89^. The combinations of nine SRAP primers that output scorable polymorphic bands were used to amplify all accessions (Table 8).

The molecular analysis of SCoT markers was done according to the procedure described by Collard and Mackill^29^. All SCoT primers were first screened using a group of all 15 samples. The nine primers that output scorable polymorphic bands were used to amplify all accessions (Table 8).

PCR amplified SCoT and SRAP fragments were detected on agarose gel based on the relative standard size (Perfect™ 100 bp DNA Ladder as standard (EURx, Poland)) using Cliqs v1.5 software (Total Lab, UK). An example of agarose gel analysis is provided in Supplementary Fig. 1. Bands were scored as 1 (present) or 0 (absent) and a binary data matrix was generated (Supplementary Table 2). Vague bands that could not be easily detected were not scored. Accessions pairwise comparisons were calculated using the Jaccard’s similarity coefficient: D_J_=2n_xy_/n_x_ + n_y_, where n_x_ is the number of bands present within accession x, n_y_ is the number of bands present within accession y, and n_xy_ is the number of bands shared by accessions x and y^90,91^. Cluster analysis based on molecular data was performed using the Jaccard’s similarity index and Ward’s clustering method with the use of the tools listed in Sect. 2.2.

Genetic relationships among the genotypes were analysed by the multiple correspondence analysis (MCA) using the R-package ‘FactoMineR’^92^. The MCA is an appropriate tool for analysing categorical data^92^. In order to investigate the efficiency of the selected primers, informativeness indices, such as the number of polymorphic bands, Resolving power (Rp), and mean PIC value, were estimated. Polymorphism information content was calculated for each primer using a simplified formula for dominant markers PIC = 1 – (p^2^ + q^2^) where p and q are the population frequency of the ith and jth allele^69,71^. The ability of the SCoT and SRAP primers to distinguish between genotypes was accessed by calculating the Resolving Power (Rp)^93^, using the formula Rp = ΣIb where band informativeness, Ib = 1 − (2 × |0.5 − p|) and p is the proportion of genotypes containing band I. To assess species-level genetic diversity R-packages ‘poppr’^94^ and ‘adegenet’^95^ were employed to compute various parameters, including the average number of observed (Na) and effective alleles (Ne)^96^, the number (NPL) and percentage (PPL) of polymorphic loci, Nei’s gene diversity/expected heterozygosity (H)^97^Shannon’s information index (I)^98^, and Nei’s coefficient of gene differentiation (G_ST_). The gene flow was then estimated using the formula Nm = ((1/ G_ST_) – 1)/4^100^. Additionally, an Analysis of Molecular Variance (AMOVA) was also performed to quantify genetic variation within and between populations for both markers using the same R packages.

A Bayesian clustering approach was employed to identify genetically similar groups of accessions, with Bayesian bar plots generated using STRUCTURE software v2.3.4^100^. The optimal number of genetic groups (K) was estimated based on 10 independent runs for each K value ranging from 1 to 10. The admixture model was applied with 100,000 Markov Chain Monte Carlo (MCMC) iterations and a burn-in period of 10,000. According to Evanno et al., the most probable K value was determined using the ΔK method^101^ via the StructureSelector web server^102^.

### Sample preparation for qualitative and quantitative analysis

*Reynoutria* spp. rhizomes were collected in the last week of October 2021 in Wroclaw (Poland) from the locations specified in Table 7. 200 milligrams of air-dried and powdered rhizomes of each sample were extracted three times with 5 mL of acetone: H_2_O (7:3 v/v) in an ultrasonic bath for 30 min at 25 °C. The extracts were separated from the plant materials by decantation after prior centrifugation. The supernatant was collected, evaporated to dryness, and lyophilized. Extracts were dissolved in 80% of MeOH to obtain a concentration of 5 mg dry weight/mL. After filtering the solutions through a 0.22 μm PTFE syringe membrane (Chromafil, Macherey-Nagel, Düren, Germany) into vials, 4 µL of the sample was injected by an autosampler into the Ultra-high performance liquid chromatography system combined with quadrupole time of flight mass spectrometry (UHPLC-QTOF-MS). All determinations were done in triplicate (biological replication meaning an independently prepared extract *n* = 3); each biological replication was measured at least twice as a technical replication.

A previously developed, validated analytical method was used to quantify the six compounds: vanicoside B, vanicoside A, resveratrol, piceid, emodin and physcion^18^. Linearity, the LOD (limit of detection), and LOQ (limit of quantification) for all quantified compounds were presented in our previous study^18^.

### Liquid chromatography–mass spectrometry analysis

Ultra-high performance liquid chromatography quadrupole time of flight mass spectrometry (UHPLC-QTOF-MS) analysis of all samples was carried out on a Thermo Ultimate 3000 RS (Thermo Fisher Scientific, Waltham, MA, USA) chromatographic system coupled to a Bruker Compact (Bruker, Billerica, MA, USA) quadrupole time of flight (QTOF) mass spectrometer, consisting of a binary pump, sample manager, column manager, and the PDA/diode array detector. Separations were performed on a Kinetex C18 column (2.1 × 150 mm, 2.6 μm, Phenomenex, Torrance, CA, USA) maintained at 30 °C. Mobile phase A (H2O: HCOOH, 100:0.1, v/v) and B (acetonitrile: HCOOH, 100:0.1, v/v) were used in a following gradient program: 0–22 min 15–50% B, 22–28 min 50–95% B, 28–33 min 95% B, 33–35 min 95 − 15% B followed by column equilibration with 15% B for 2 min between injections. The flow rate was 0.3 mL/min. Analysis of all samples was repeated at least two times as consecutive injections. UV-Vis spectra were recorded in the range of 200–450 nm. Chromatograms were acquired at 298 nm. High-resolution quadrupole time-of-flight mass spectrometer was equipped with electrospray ionization (ESI-HR-QTOF-MS). ESI-MS conditions were as follows: splitless, nebulizer pressure 30 psi; dry gas flow 8 L/min; dry temperature 250 °C; and capillary voltage 2.2 kV for negative ion mode and 4.5 kV for positive ion mode (for physcion). Mass spectra were recorded using scan range (m/z) of 50–2200. The collision energy was set automatically from 20 to 40 eV, depending on the m/z of the fragmented ion. The identification of constituents found in plant materials was based on MS^1^ (accurate mass) and MS^2^ spectra which were compared with a library.

For statistical analysis raw data obtained from UHPLC-QTOF-MS (DataAnalysis 4.2, Bruker) analysis were converted by MSConvert (ProteoWizard) to mzML format.

Next, all data were uploaded into the XCMS Online platform (https://xcmsonline.scripps.edu/landing_page.php?pgcontent=mainPage). The single job analysis was chosen. All parameters selected for processing the job are presented in Supplementary Table 3. Result table with features (after reducing their number by removing those with the lowest intensity to 117) were used for statistical analysis. Table with features is presented in the Supplementary Table 4. Among the 117 features included in the statistical analysis, most were tentatively identified.

## Electronic supplementary material

Below is the link to the electronic supplementary material.

Supplementary Material 1

Supplementary Material 2

## Data Availability

Morphological and molecular data generated and analyzed during this study are included in this published article and its supplementary information files. The UHPLC-QTOF-MS raw data generated and analyzed in this study are available from the corresponding author on reasonable request.
