# Supplementary material for: Integrative morphological, phytochemical, and molecular identification of three invasive and medicinal Reynoutria species
Source: Sci Rep. 2025 Feb 18;15:6001. doi: 10.1038/s41598-025-90494-2 (PMC11836466; doi:10.1038/s41598-025-90494-2)
Supplement: Supplementary file 2 — Supplementary Material 2 [file 41598_2025_90494_MOESM2_ESM.docx]

**Integrative morphological, phytochemical, and molecular identification of three invasive and medicinal *Reynoutria* species.**

**Marta Stafiniak^1^, Monika Bielecka^1^*****, Krzysztof Kujawa^2^, Anna Jezierska-Domaradzaka^1^, Bartosz Pencakowski^1^, Aleksander Basiak^3^, Adam Matkowski^1^, Izabela Nawrot-Hadzik^1^**

^1^Department of Pharmaceutical Biology and Biotechnology, Wroclaw Medical University, Wroclaw, Poland

^2^Statistical Analysis Centre, Wroclaw Medical University, Wroclaw, Poland

^3^Department of Pediatrics, Endocrinology, Diabetology and Metabolic Diseases, Wroclaw Medical University, Wroclaw, Poland

*monika.bielecka@umw.edu.pl


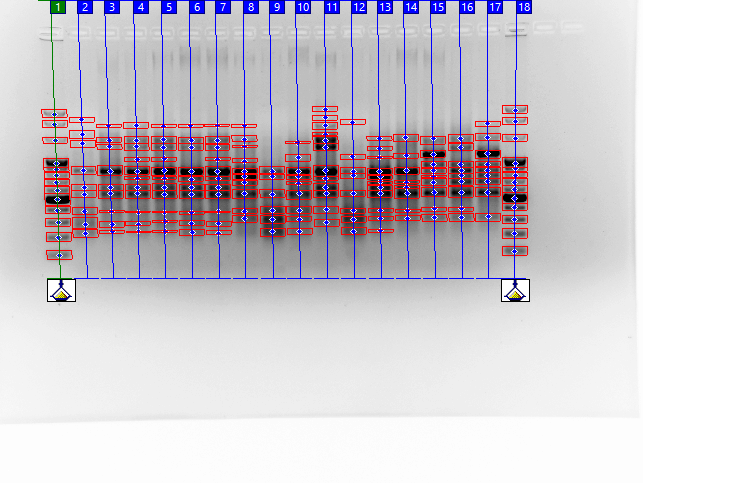

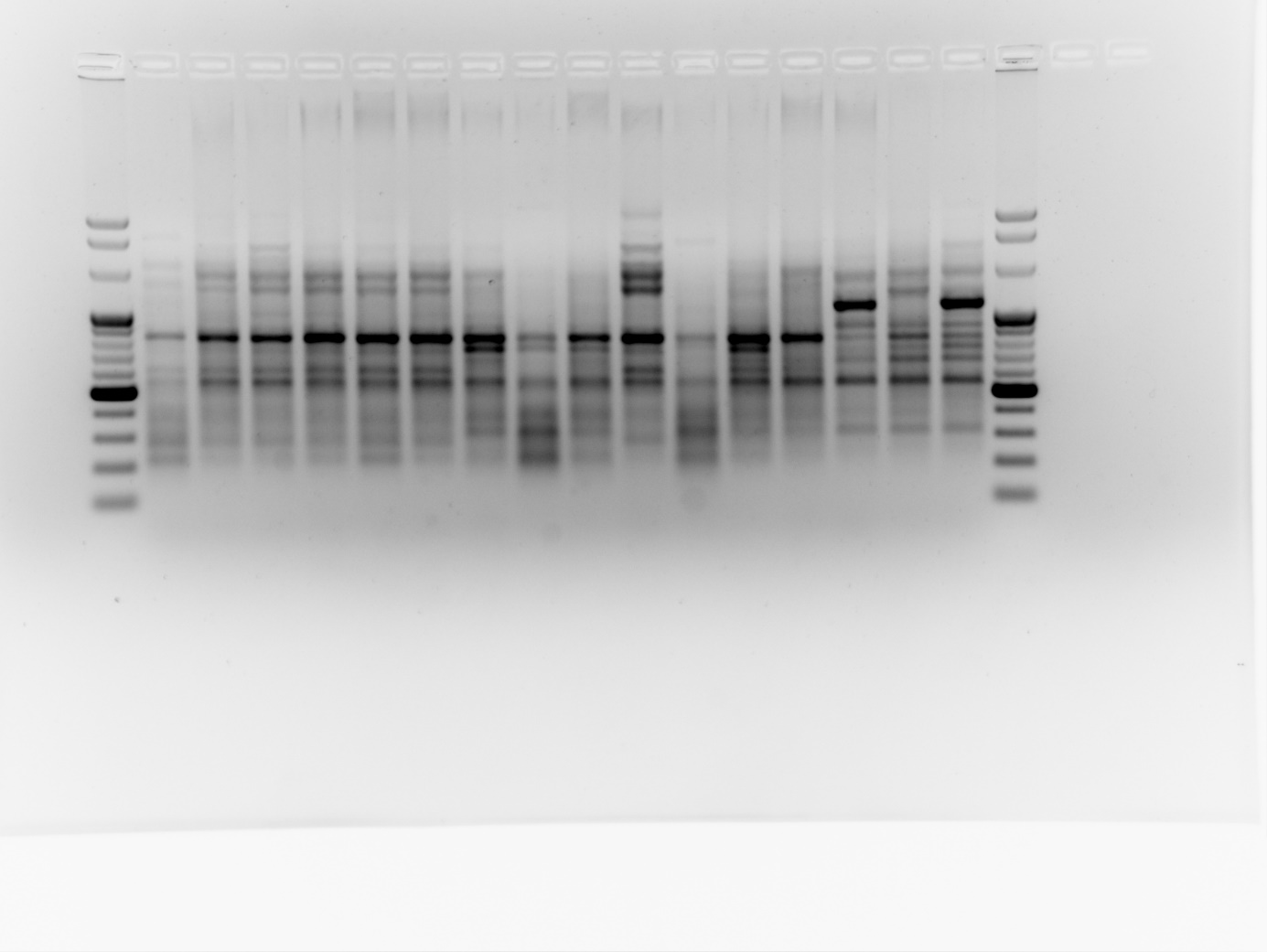


A

B


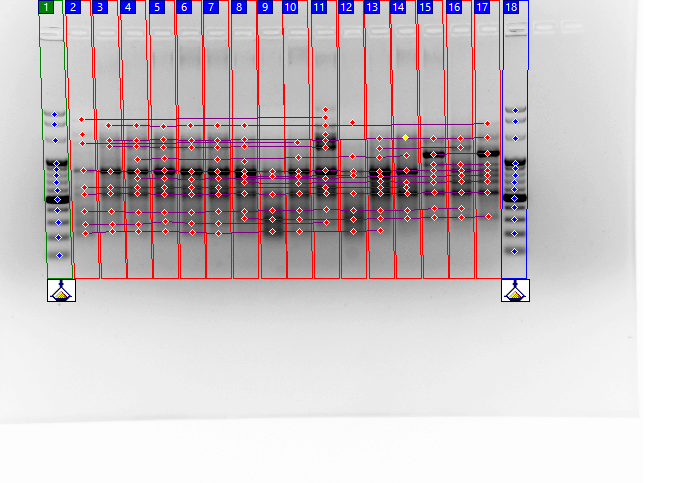


C

**Supplementary Figure 1** Band analysis workflow. Amplification profile of SCoT1 marker in Reynoutria populations (A); Bands detection with Cliqs v1.5 software (Total Lab, UK) (B); Aligning visible bands and matrix creation (C); Left and right-most lane – Perfect™ 100 bp DNA Ladder (EURx). Lanes 2 to 7 (locations RJ2, RJ5, RJ6, RJ8, RJ10, RJ31) – R. japonica; lanes 8 to 14 (locations RB12, RB13, RB14, RB15, RB17, RB18, RB20) – R. x bohemica, lanes 14 to 16 (locations RS21, RS22, RS23) – R. sachalinensis (RS).


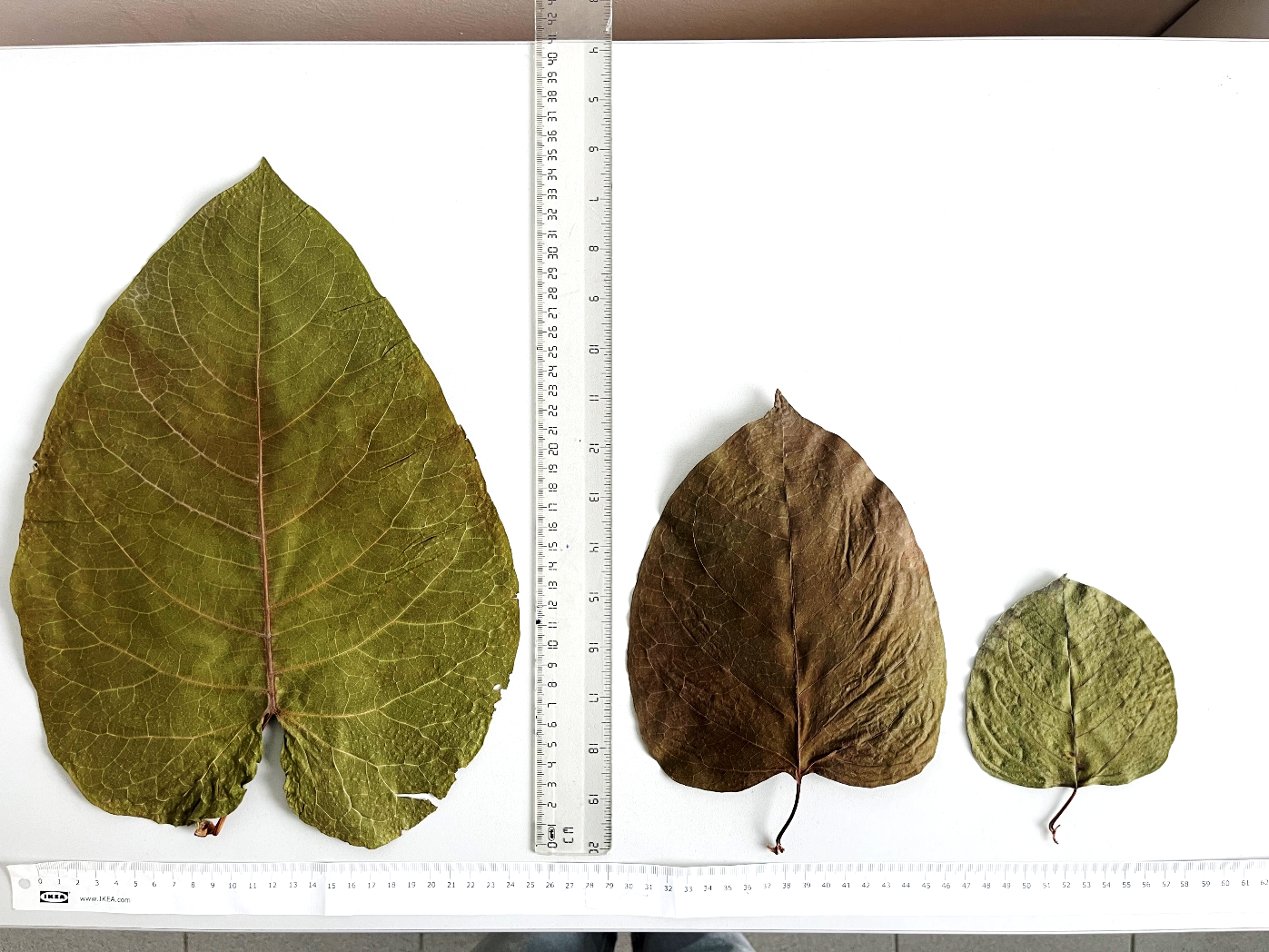


C

A

B

**Supplementary Figure 2** Variation of leaves size and morphology in typical representatives of R. sachalinensis (A), R. x bohemica (B), R. japonica (C).

**Supplementary Figure 3** Chromatograms of extracts obtained from samples from different locations, as specified in Table 6 – R. japonica (red), R. x bohemica (blue), R. sachalinensis (green).
